# Factors Determining Quality of Care in Family Planning Services in Africa: A Systematic Review of Mixed Evidence

**DOI:** 10.1371/journal.pone.0165627

**Published:** 2016-11-03

**Authors:** Gizachew Assefa Tessema, Judith Streak Gomersall, Mohammad Afzal Mahmood, Caroline O. Laurence

**Affiliations:** 1 School of Public Health, The University of Adelaide, Adelaide, Australia; 2 Department of Reproductive Health, Institute of Public Health, University of Gondar, Gondar, Ethiopia; Liverpool School of Tropical Medicine, UNITED KINGDOM

## Abstract

**Background:**

Improving use of family planning services is key to improving maternal health in Africa, and provision of quality of care in family planning services is critical to support higher levels of contraceptive uptake. The objective of this systematic review was to synthesize the available evidence on factors determining the quality of care in family planning services in Africa.

**Methods:**

Quantitative and qualitative studies undertaken in Africa, published in English, in grey and commercial literature, between 1990 and 2015 were considered. Methodological quality of included studies was assessed using standardized tools. Findings from the quantitative studies were summarized using narrative and tables. Client satisfaction was used to assess the quality of care in family planning services in the quantitative component of the review. Meta-aggregation was used to synthesize the qualitative study findings.

**Results:**

From 4334 records, 11 studies (eight quantitative, three qualitative) met the review eligibility criteria. The review found that quality of care was influenced by client, provider and facility factors, and structural and process aspects of the facilities. Client’s waiting time, provider competency, provision/prescription of injectable methods, maintaining privacy and confidentiality were the most commonly identified process factors. The quality of stock inventory was the most commonly identified structural factor. The quality of care was also positively associated with privately-owned facilities. The qualitative synthesis revealed additional factors including access related factors such as ‘pre-requisites to be fulfilled by the clients and cost of services, provider workload, and providers’ behaviour.

**Conclusion:**

There is limited evidence on factors determining quality of care in family planning services in Africa that shows quality of care is influenced by multiple factors. The evidence suggests that lowering access barriers and avoiding unnecessary pre-requisites for taking contraceptive methods are important to improve the quality of care in family planning services. Strategies to improve provider behavior and competency are important. Moreover, strategies that minimize client waiting time and ensure client confidentiality should be implemented to ensure quality of care in family planning services. However, no strong evidence based conclusions and recommendations may be drawn from the evidence. Future studies are needed to identify the most important factors associated with quality of care in family planning services in a wider range of African countries.

## Background

Strengthening family planning services is crucial to improving health, human rights, economic development, and slowing population growth [1]. Yet, globally more than 289,000 maternal deaths occurred in 2013 of which nearly 99% (286,000) women died in developing countries, of which a larger proportion were African countries [2]. Studies have showed that up to 40% of maternal deaths could have been averted through use of family planning services [3,4]. In 2015, 64% of married or in-union women of reproductive age were using some form of contraception in the world but the use was much lower in Africa (33%) [5]. It is estimated that globally, 225 million women who want to avoid pregnancy are not using safe and effective family planning methods [6,7]. Most of the women with an unmet need for contraceptives live in 69 of the poorest countries [8]. This unmet need is due to both rapidly growing populations and shortage of family planning services [6,7].

In response to this, increasing access to family planning services has become a globally recognized public health priority. A number of global partnerships such as the International conference on Population and development (ICPD) in 1994 [9], the Millennium Development Goal (MDG) summit in 2000 [10], and the London Summit on Family Planning in 2012 endorsed a global partnership known as Family Planning 2020 (FP2020). This partnership aims to enable 120 million more women and girls to use contraceptives by 2020 in 69 of the world’s poorest countries [11].

Improving the quality of care in family planning services is key to improve use of family planning services in developing countries, both by attracting new contraceptive users and by maintaining existing users (i.e. ensuring continued engagement with services) [12–19]. Providing decision makers in developing countries, including in Africa, with the best available evidence on the factors that determine the quality of care in family planning services, from the perspective of clients and health care providers, is important to inform the design and implementation of the most effective, efficient and acceptable measures.

The World Health Organization (WHO) defines family planning as “the ability of individuals and couples to anticipate and attain their desired number of children and the spacing and timing of their births. It is achieved through use of contraceptive methods and the treatment of involuntary infertility” [20]. A report developed by Center for Disease control (CDC) and the Office of Population Affairs (OPA) of the United States Department of Health and Human Services articulates family planning services broadly in terms of infertility treatment and sexually transmitted disease (STD) screening and treatment, pregnancy testing and counseling services, helping clients who want to conceive; providing preconception health services besides services related to contraceptive provision and counseling [21]. However, previous studies conducted to assess the quality of care in family planning services viewed family planning services narrowly, as the provision or prescription of contraceptive methods after women receive counseling on contraception to help them delay or prevent pregnancies [22–24].

Literature relating to quality of care in family planning services found that a number of approaches have been used to define and measure quality of care in family planning services and its determinants, and these vary with the stakeholders’ priorities and perspectives [25]. The Donabedian [26,27] and Bruce Frameworks [28] are the two conceptual frameworks that have been used most frequently since the early 1990s to inform empirical work assessing the quality of family planning services and factors that determine quality of care in family planning services. The latter framework was developed specifically for family planning services.

Donabedian [27]p5 defined quality of care as “the application of medical science and technology in a manner that maximizes the benefits to health without correspondingly increasing the risk”. This model, developed in 1988, was intended to assess quality of care in various health services, including family planning. He described quality of care in a linear model comprising the three components—structure, process, and outcome [26]. This model has continued as a dominant paradigm for assessing quality of care in health services although it has been critiqued for failing to incorporate precursors to quality care such as patient characteristics and broader environmental factors (including the patient’s cultural, social and political context), as well as factors related to the health profession itself [29].

Underpinned by the work of Donabedian, Bruce and Jain [28,30] identified six elements of quality of care in family planning programs that “reflect the six aspects of services that clients experience as critical” [28]p63. These six elements were: choice of methods; information given to clients; technical competency of providers; interpersonal relations; follow-up mechanisms; and appropriate constellation of services [28]. The ‘choice of methods’ refers to having a range of contraceptive methods offered to the clients considering their diverse needs such as clients age, gender, contraceptive intention and lactation status. ‘Information given to clients’ refers to the information provided to clients during service interactions that enables clients to choose and use contraception with competence and satisfaction. This includes information about a range of available contraceptive methods, method contraindications, method advantages and disadvantages, how to use selected method, potential side effects, and continuing care from service providers. The ‘technical competence’ aspect involves providers’ clinical techniques, use of protocols, and implementing aseptic procedures in performing clinical conditions. ‘Interpersonal relations’ refer to the degree of empathy, trust, assurance of confidentiality, and sensitivity of providers to meet the client’s needs and expectations. The ‘follow-up mechanism’ considers how service providers encourage clients on the continuity of use through well-informed mechanisms such as community mass media, client-based follow-up mechanisms (return appointments), or home visits. The last component, ‘appropriate constellation of services’, is suitability of family planning services in terms of their location being at convenient place and time and the level of integration with other reproductive and maternal health services. Since its development, this framework has been widely used to inform studies measuring quality of care in family planning services [31–34].

The Donabedian and Bruce/Jain Frameworks have identified a range of outcomes for quality of care including client satisfaction, change in behaviour and contraceptive knowledge, reduction in fertility and mortality [26,28]. However, measuring some of these outcomes are complex, time consuming, and expensive. To address this challenge, client satisfaction, a simple and more practical outcome measure, can serve as a good indicator and outcome measure in resource limited countries [35]. Client satisfaction has been found as a key determinant of uptake and continued use of family planning services [12,36]. Measuring client satisfaction not only evaluates certain aspects of quality of care but also indicates better prospects for sustainability in terms of recruiting new users and maintaining those clients who are already in the service [12,36,37]. Evidence has also showed that good quality of healthcare positively correlates with patient satisfaction [38]. As a result, client satisfaction is widely used for measuring quality of care in family planning and other health services and has been used in a number of previous studies in low and middle income country settings aimed at determining the factors associated with quality of care in family planning services [22,24,39–41]. Therefore, client satisfaction was used as an outcome of interest in the quantitative part of this review.

A preliminary search of databases found no existing systematic review, or systematic review protocol, with the objective of identifying factors determining the quality of care in Africa or in any one or group of African countries. The objective of this systematic review was to identify and synthesise quantitative and qualitative evidence to understand factors determining the quality of care in family planning services in Africa.

## Methods

This review followed best practice guidelines for systematic review of quantitative and qualitative evidence [42]. The review was based on published protocol [43] and followed established Preferred Reporting Items for Systematic Reviews and Meta-Analyses (PRISMA reporting guidelines (S1 Table) [44].

### Inclusion criteria

Quantitative and qualitative African studies, of all design types, published in peer-reviewed journals and grey literature, between 1990–2016 were considered. The year 1990 was set as the start date for the search because this was when quality of care began to be emphasized in family planning services [28,45,46].

The participants of the studies were clients and/or providers of family planning services. Female and male clients and providers of all ages, any socio-economic status and from all ethnic and language groups from Africa were considered. Clients and providers of all levels (lower levels such as health post or higher levels such as tertiary hospitals) and types (public or private) of health service facility types from Africa were considered. Family planning services were defined as provision or prescription of contraceptive methods after women receive counseling on contraception to help them delay or prevent pregnancies.

For the quantitative component of the review, the exposure of interest was factors that were associated with quality of care in family planning services. An exposure factor was identified when a study reported a statistically significant association between the exposure (independent) and the outcome (dependent) variable. Studies that investigated factors including facility, client, and provider characteristics associated with quality of care in family planning services in Africa were considered. The outcome of interest for the quantitative component of the review was quality of care in family planning services. While there are a number of outcomes that could be used to measure the quality of care of family planning services such as knowledge and behavioral change, fertility reduction and mortality reduction [26,37], as described in the background section, client satisfaction was selected as a proxy outcome measure for this review. In the included studies, client satisfaction was assessed in three ways: First, using proxy questions such as satisfaction on waiting time, privacy for not being seen or heard by others, availability of family planning methods, cleanliness of the facility, costs of the services, the staff treatment. Then studies developed one aggregate variable using principal component analyses to present the measure as a continuous variable or dichotomized in to binary variable as satisfied or not satisfied; Secondly, using a Likert scale in ten categories with the higher scale indicating greater satisfaction and then creating a binary variable using the mean as a cut point (i.e those who scored below the mean regarded as less satisfied while those who scored above the mean regarded as highly satisfied. Thirdly, using client’s overall satisfaction and then created a binary outcome comprising as satisfied and not satisfied.

In the qualitative component of the review, the phenomenon of interest was client and provider experiences and/or perceptions of the factors that determine quality of care in family planning services.

### Search and study selection

We followed a three-staged process for a comprehensive search strategy of electronic sources [42]. In addition, one researcher known to the lead reviewer working in family planning and reproductive health services research in Ethiopia was contacted through email to identify any other relevant studies.

The databases searched were PubMed, CINAHL, EMBASE, Scopus, POPLINE, the Cochrane Collaboration reports of controlled trials (CENTRAL), African Index Medicus (AIM), and Web of Science using the search strategy and key terms outlined in the S2 Table. The search engines Google, Google Scholar, and specific websites such as World Bank, WHO, Family Health International, International Planned Parenthood Federation (IPPF), and the Demographic and Health Survey program were searched for grey literature.

An overarching search strategy was developed by GAT in consultation with JSG, CL, AM and then Librarian was consulted for specific approach on how to execute. The search terms used were: (“quality of care” OR “quality of health care” OR quality) AND (“family planning” OR “family planning services” OR “contraceptive services” OR “birth control services” OR contraceptive OR contraception) AND Africa (S2 Table).

Citations identified through the search strategy were initially reviewed for inclusion based on information contained in titles, abstracts, citation information, and keywords. One reviewer (GAT) screened the records to determine eligibility. Full text articles were obtained for all eligible studies and for those requiring further review to determine eligibility. Articles on full text examination that did not match the inclusion criteria were excluded, and the reasons for exclusions were noted (S1 Fig and S1 Text). Those articles that fulfilled the inclusion criteria were critically appraised and included in the review.

### Assessment of methodological quality

Two reviewers (GAT and JG) independently appraised the methodological quality of the studies that met the inclusion criteria. Quantitative studies were appraised using the tool for appraisal of quantitative descriptive studies in the Meta-Analysis of Statistics Assessment and Review Instrument (JBI-MAStARI) [42]. Qualitative studies were appraised using the appraisal tool in the JBI Qualitative Assessment and Review Instrument (JBI-QARI) [42]. Studies scoring greater or equal to seven were deemed high quality, those scoring four to six were deemed of medium quality and those scoring less than four were deemed low quality for both instruments.

### Data extraction

Data extraction was performed using templates based on the JBI-MAStARI data extraction tool for quantitative data and JBI-QARI for the qualitative studies [42]. For each quantitative study, gathered information was gathered on the general characteristics of the study (type of study, aim, country, methodology), and on statistically significant factors identified by the included quantitative studies. In addition to the general study characteristics, the experiences and views of clients and providers were extracted from qualitative studies as well as the author’s interpretation of findings along with illustrations (participant’s voices). The findings and supporting illustrations extracted from the included qualitative studies are provided in S3 Table.

### Data synthesis

Due to quantitative studies’ heterogeneity in assessing the outcome variable, we performed textual narrative analysis after tabulating individual quantitative studies in terms of their characteristics, key significant factors, and conclusions of the individual studies [42]. The qualitative findings were synthesized using meta-aggregation [42]. Two syntheses of the perceptions were performed, one using the clients’ experiences and perceptions of quality of care, the other the provider experiences and perceptions. In each segregated synthesis, the pooled findings were first grouped into categories defined by their similarity of meaning and then combined into one or more synthesized finding(s) that captured their meaning. All the findings extracted from the qualitative studies meeting the inclusion criteria were judged credible and used in the syntheses (S3 Table).

## Results

### Description of studies

In total, 4334 potentially relevant records were identified, of which 3780 remained after removing duplicates. Title and abstract screening led to an additional 3682 exclusions. A total of 98 articles were deemed eligible for full text analysis, of which 87 articles were excluded for not meeting the inclusion criteria. (S1 Text lists the articles excluded on full text examination with the reasons for exclusion). A total of 11 studies [22,41,47–55], eight quantitative [22,41,47–50,54,55] and three qualitative [51–53], met the inclusion criteria.

Of the eight quantitative studies [22,41,47–50,54,55] reporting the factors associated with quality of care in family planning services, four [22,41,49,50] undertook secondary data analysis, with two studies [22,50] undertaking analysis in three different countries (three countries each). Two studies [22,50] identified the factors for higher level (Hospital/health centre) and lower level (clinics/others) facilities separately. Two studies were conducted in Egypt, three in Kenya, two in Senegal, two in Ethiopia, one in Ghana, one in Tanzania, and one in Namibia (Table 1). Two studies [22,49] employed the same database from Senegal but used a different study population and data analysis techniques. In total, 3219 health facilities and 7676 clients were included in the eight quantitative studies.

**Table 1:** Characteristics of the quantitative studies included in the review.

General article information								Quality score
First author, year of publication, and reference number	Aim(s) and study design	Country and year of study	Study Population and sample size	Data collection method(s)	Outcome measurement	Data analysis	Limitations identified by the author(s)	
Abdel-Tawab 2002[47]	• Aims: to examine the feasibility, acceptability, and effectiveness of client-centred models in FP clinics • Design: Cross sectional study	Egypt 1992	• Female family planning client (n= 112) • Mean age= 29 years (range 19-45) • 84% rural • Physician (n= 34) • Mean age = 32 years (range 27-50) • Family planning clinics (n= 31)	Client exit interview, audiotaped data for provider-client interaction data, physician interview	• Client satisfaction was assessed by considering five proxy questions which were rated 0 to 10, with higher score indicating greater satisfaction. • An average satisfaction score (mean score) was calculated and client satisfaction was dichotomized as highly satisfied and less satisfied.	Multivariate logistic regression analysis	No limitation information was provided	Moderate
Agha 2009[41]	• Aim: to compare the quality of family planning services delivered at public and private facilities • Design: Secondary analysis of cross-sectional study	Kenya 2004	Health facilities (n= 323) and family planning clients (n= 628) in a subset of 172 facilities.	Facility inventory, observation, and client exit interviewing	Client satisfaction was assessed through proxy questions and those clients who responded ‘no problem’ to these questions were regarded as satisfied client and otherwise taken as not satisfied.	Multivariate logistic regression analysis	Sample for private facilities was smaller	Moderate
Hutchinson 2011[50]	• Aim: to quantify the differences in the quality of family planning services at public and private providers in three countries • Design: secondary data analysis of cross sectional studies	• Tanzania 2006 • Kenya 2004 • Ghana 2002	• Tanzania: Health facilities (n=482), providers (n=1244), and clients (1005) • Kenya: facilities (n=323), providers (n=860), clients (n=628) • Ghana: facilities (n=386), providers (n=845), clients (n=611)	Data collected through facility survey, observation and client interview	Client satisfaction was measured in two ways. Responses were dichotomized as satisfied if there were ‘no problem’ in proxy questions related to client satisfaction. Additionally, they calculated index of satisfaction using principal component analysis and took as a continuous variable. Factors were identified using both measurements.	Both multivariate linear regression and multivariate logistic regression analysis conducted. The regression analysis were conducted for hospital and clinics separately.	Inability to distinguish between for-profit and not-for-profit private facilities	Moderate
Tafese 2013[55]	• Aim: to assess the quality of family planning services in primary health care centres • Design: Cross-sectional study	Ethiopia 2011	Family planning clients (n=301) mean age (SD)= 26(+5), range (15-45), 61.5% were from rural Health centres (n=5)	Exit-interview of women at facility, and observation of provider-client interactions	Client satisfaction measured through 10 proxy questions and the principal component analysis was used to create an aggregate measure of continuous variable. Each included proxy question was assessed using a 5-points Likert scale (0 to 5)	Multivariate linear regression	Hawthorne effect during provider observation, Courtesy bias during the exit interview and introduction of observer bias	High
Wang 2014[22]	• Aim: to assess the quality of care at health facilities in providing family planning, antenatal care and sick child care • Design: Cross sectional study	• Kenya 2010 • Namibia 2009 • Senegal • 2012/2013	• Kenya: Health facilities (n=575), providers(n=1583), clients (n=1004) • Namibia: • Facilities(n=362), providers (n=966), clients (n=983) • Senegal: • Facilities (n=338), • providers (n=735), clients (n=968)	In all the three countries, data were collected through facility inventory assessment, client exit interview, provider-client interaction observation, provider interview	Client satisfaction variable was rated as an index of problems encountered during the visit (none versus any). Client’s responses for these proxy questions were then aggregated into an index using principal components analysis	Multivariate linear regression	Observer bias and social desirability bias	Moderate
Assaf 2015[49]	• Aim: to examine the quality of care in health facilities in Senegal, with a focus on family planning services • Design: Secondary data analysis of cross-sectional study	Senegal 2012/13and 2014	• Two rounds • Round 1, facilities (n=364), clients (n=872), and provider (n=872) involved • Round 2, facilities (n=363)	Data collections was made in two rounds. Facility inventory survey, observation of provider-client interaction, and providers interviewing were made in each survey periods	Client satisfaction was measured based on a general question about overall client satisfaction on the family planning services. The categories of the responses were very satisfied, more or less satisfied, and not satisfied. Finally they created a binary variable as very satisfied or not satisfied.	Multivariate logistic regression	Social desirability bias, client satisfaction maybe over-reported	Moderate
Argago 2015[48]	• Aim: To assess client satisfactions with family planning services and associated factors • Design: cross-sectional study	Ethiopia 2014	• Family planning clients (n= 324) • Mean age= 28 years (SD=5.57), range (17-42) • 72.8% were repeated users. • Health facilities (n=20)	Client exit interview was conducted	Client satisfaction score was calculated using 18 proxy questions and then binary variable was devised as low and high satisfaction.	Multivariate logistic regression	The study was solely based on client’s information. It did not include provider-client observation or a facility inventory assessment.	Moderate
Nasr 2016[54]	• Aim: to assess the association between quality of family planning services and client satisfaction level • Design: Cross-sectional study	Egypt 2014	• Clients of family planning (n=240) between 20-40years • Mean age=31.6 years (SD= 7.0) • Women at least 2 children • Health facilities (n=10) • Nurses (n=20) • Mean age=36.4 (SD=8.04) • 10 (50%) had less than 5 years of work experiences	Facility survey, observation, and client interview	Client satisfaction measured through Likert scale and then binary outcome variable was created as satisfied and not satisfied.	Chi-square test	No limitation information was provided	Moderate

SD- Standard Deviation

Three qualitative studies [51–53], two conducted in Kenya [51,52] and one in Uganda [53] met the eligibility criteria of the qualitative review component. One [52] included client and provider participants, one [51] had only client participants, and one [53] had provider participants. In total, there were 122 client and 65 health provider participants in the three qualitative studies (Table 2).

**Table 2:** Key characteristics of qualitative studies included in the review.

First author, year of publication and reference number	Aim(s) of the study	Country and year of study	Study Participants and sample size	Data collection method (s) and analysis	Limitations identified by the author(s)	Quality Score
Lewis 1995[52]	To define the laypersons' and providers' dimensions of quality of care and compare them with the Bruce-Jain elements.	Kenya 1994	Women 15-49 years (N=31); Service providers (n=17), simulated clients (n=51)**, Clinics (n=9), 2 urban and 2 rural setups	• FGD* with clients • In-depth interviews with clients, simulated client visits**, indepth interview with provider and managers Services delivery points’ visits • Analysis: Thematic analysis	No limitation information was given	Moderate
Mugisha 2008[53]	To assess providers’ perceptions of quality of care and the barriers to quality services at the organizational and societal levels.	Uganda 2002	• Service providers and managers (n= 38, midwives=33; nurses=6) • Almost half of the providers were aged between 31 and 45 years and most were married.	• FGD* • Provider and manager interviews • Analysis: not explicitly described but thematic analysis seemed to be employed.	No limitation information was given	High
Keesara 2015[51]	To describes women’s expectations and experiences when seeking contraceptive care from private and public facilities in Nairobi.	Kenya 2013/2014	Postpartum reproductive aged women (n= 91)	• FGD* and • In-depth interview with clients • Data analysis: thematic analysis	• Participants lived far away from public facility were not included. • The type of private facility that the interviewee had attended was not differentiated. • Social desirability bias.	High

* FGD- Focus Group Discussions

**those findings from simulated clients were not included in this analysis

Of the eight quantitative cross-sectional studies [22,41,47–50,54,55], seven were rated as moderate quality and one was rated high quality. Two [48,55] out of eight studies undertook systematic random sampling in selecting their study participants. The samples taken for the study were representative and outcomes were measured using reliable methods. Most (7/8) of the studies assessed their outcome using objective measures through proxy questions. Most (7/8) studies controlled confounding factors using multivariate regression analysis. Most (7/8) studies did not describe those participants who withdraw or refused to participant in the study. This would affect the studies generalizability when they had a high rate of non-response and eventually influence generalization in the present review.

Of the three qualitative studies, two [51,53] were assessed as high quality, receiving scores of 7/10 and one [52] was assessed moderate quality (6/10). All three of these studies lacked description of the congruency between the philosophical perspectives and research methodology used. Failure to describe how the researchers’ perspectives may have influenced the analysis and interpretation of findings was identified as the main weakness in the three qualitative studies, potentially undermining credibility (S4 Table).

### Factors associated with quality of care in family planning services identified by the quantitative evidence

Eight quantitative studies [22,41,47–50,54,55] identified various factors determining quality of care in family planning services in seven African countries. Table 2 shows the factors that the studies have found to be statistically significantly associated with quality of care, categorized into factors related to the client, provider, facility, structure, and process. These factors were related to the demographics of clients, the provider involved in the provision of family planning clients, and the general characteristics of the health facilities in terms of locations, ownership. In addition, those factors related to the infrastructure, equipment, and provider-client interaction were classified as structural and process related factors.

#### Client, provider, and facility factors

Two studies [41,47] showed that the age of the client was associated with client satisfaction. However, the effect of age was inconsistent in that Abdel-Tawab et al. [47] found young clients were less likely to be satisfied with family planning services while Agha et al. [41] revealed young clients as more likely to be satisfied than their older counterparts. Another three studies [22,49,55] showed no statistically significant association between age of clients and client satisfaction.

A significant association between client’s educational status and quality of care in family planning services was found in three studies [41,50,55]. Clients with higher educational levels were identified as more likely to be satisfied with quality in two studies [50,55], while, one study showed less educated women as more likely to be satisfied [41]. One study revealed that repeat family planning clients were more likely to be satisfied with the service than first time clients [48] (Table 2).

With respect to the provider variables, provider’s years of education and number of years of experience were significantly associated with client’s satisfaction in family planning services [41,49]. One study also showed that clients were less satisfied with family planning services provided by young physician than when they received services from older physicians [47] (Table 2).

Regarding the health facility characteristics [22,41,49,50], firstly, client satisfaction was reported in three studies as greater for private than publicly owned health facilities [22,41,50]. Secondly, client satisfaction was found in two studies as being associated with geographic location of health facilities [41,49].

#### Structural factors

Health facility’s structural factors such as staffing levels, management, availability of materials and equipment were found to be associated with the quality of care in family planning services [22,41,48,50,54]. There was a positive but weak association between number of staff in facilities and client’s satisfaction in that clients were more likely to be satisfied in facilities possessing higher staff numbers [41]. Greater numbers of days in a week for family planning service, facilities closer to the client’s residence, and with what client perceived to be convenient opening hours were positively associated with client’s satisfaction in family planning provision [48,50]. In contrast, Wang et al. [22] found that greater number of days in the week for family planning services provision did not result in client satisfaction In addition, two studies showed facility cleanliness as a factor associated with client’s satisfaction [48,54] (Table 3).

**Table 3:** Summary of the statistically significant factors affecting quality of care in family planning services in Africa.

First Author, year of publication and reference number	Factors for QoC in FP services				Authors conclusions
	Socio-demographic and other factors*	Structural Factors*	Process Factors*	Controlled variables during multivariate analysis	
Abdel-Tewab 2002[47]	Client’s age less than 35 (AOR=0.3), physicians age less than 35 (AOR=0.2)		Client-centred communication (AOR= 2.8), high positive talk by physician (AOR= 2.0), FP methods chosen by the client (AOR=3.3)	Physician’s duration of stay in the project, Physician’s attendance on the training course focusing on counselling and interpersonal communication, types of FP used, Physicians to clients talk ratio.	Client-centred communication was associated with a three-fold increase in the likelihood of client satisfaction. In addition, solidarity statements by the physician (positive talk) was also important for client satisfaction.
Agha 2009[41]	Private facility (AOR=3.1), Hospital (AOR=0.4), Region: Central province (AOR=8.9), coast (AOR= 0.2), client’s age 25-34(AOR=0.4), 34+ (AOR=0.07), client’s primary education (AOR=0.07), secondary education (AOR=0.008)	Index of services availability (AOR=1.7), number of staff per facility (AOR=1.002), providers with 7+ years of experience (AOR=3.9), providers received family planning training in last 3 years (AOR= 3.6), providers believed supervisor support would help improve (AOR= 4.6), provider believed incentives would help improve services (AOR=3.1), Provider believed there was opportunity for promotion (AOR= 3.1), clients paid for family planning (AOR=0.4)	Confidentiality assured (AOR= 1.8), high reproductive history and physical examination score (AOR=1.2), longer waiting time (AOR=0.98),	• Catchment population, • Time taken to reach facility	Client satisfaction is much higher at private facilities. Technical quality of care provided is similar in public and private facilities.
Hutchinson 2011[50]	• **Ghana** • NGO facilities (β1=0.3034) (H), (β2=0.7329) (C), • Client education: primary (β2= 0.5967), secondary (β2=0.8252) (H) • **Kenya** • NGO facilities (β1 =0.6930) (C), • Urban facility (β1= -0.8163) (C) • **Tanzania** • NGO facilities (β1=1.1462), (β2=2.4378) (C)	• **Ghana** • Supervisory visit in last 6 months (β2=-1.1562) (H) • Number of days FP offered (β2= 0.4559) (H) • Quality stock inventory (β2=0.4317) (H) • **Kenya** • Facility inventory (β1=0.1243) (C), trained provider present 24 hours (β2=0.7691) (C), supervisory visit in last 6 months (β1= -0.3453) (H), (β2 = -1.4670) (C), total FP offered (β1= -0.0839) (H), FP client record maintained (β1= -0.3421) (H), (β2= 1.1700) (C) number of trained provider (β1= -0.1385) (H) • **Tanzania** • Facility inventory (β2= 0.1091), (β1=0.0628) (H), protocol on FP followed (β1= 0.1376)	• **Ghana** • Number of reproductive health related questions asked and physical exam done (β1= 0.0308) (C), • Client told about side effect (β1= 0.5430) (C)(β1= 0.3884), • Injection method prescribed (β1=0.3884) (C), long waiting time (β2= -0.0048), (β1=-0.0021) (H) (β2= -0.009), (β1= -0.0037) (C) • **Kenya** • Number of reproductive health related questions and physical exam (β1= 0.0431) (H), (β2= 0.1418) (C) • confidentiality assured (β1= 0.4389) (β2=0.3512) (H), long waiting time (β2= -0.008), (β1=-0.004) (H), (β2= -0.011) (C) • **Tanzania** • Injection method prescribed (β2=0.5246), • Long waiting time (β2= -0.007) (β1=-0.0030) (H) (β1=0.0237) (C)	In the three countries, the catchment population, structural factors such as number of staff, system for quality assurance, number of FP trained and process factors such as visual and auditory privacy, client concerns noted were controlled	Private health facilities appear to be of higher (interpersonal) process quality than public facilities Client satisfaction appears considerably higher at private facilities
Tafese 2013[55]	Educational status (β2=0.09)		Perceived sufficiency of consultation** (β2=0.24), perceived facilitated services***(β=0.17),	Marital status, preferences of additional children, discussion with husband/partner, occupational status, religion, age, and waiting time	• There was lack of critical resources for the provision of quality family planning services. • Client satisfaction was affected by recipient of adequate information about the chosen family planning method, and educational status
Wang 2014[22]	• **Kenya** • Government managed facilities (β1=-0.28) (C) • **Senegal** • Government managed facilities (β1= -0.68) (H)	• **Namibia** • Supervisory visit to facility within the past 6months (β1=0.27) (C) Number of days FP services provided (β1= -0.041) (C) • Number of FP visual aids (β1= -0.06) (C) • **Senegal** • Supervisory visit to facility within the past 6months (0.83) (H) • Number of days FP services provided (β1= -0.14) (C)	• **Kenya** • Process composite score (β1=0.09) • Injectable provided/prescribed (β1=0.47) • Waiting time (β1=-0.01) (H) (β1=-0.00) (C) • **Namibia** • Waiting time (β1= -0.01) (H) (β1=-0.021) (C)	Clients age, Educational status	• The client satisfaction score was higher at clinics and other types of facilities than hospitals/health centres in Senegal. • Process attributes seem to be more predictive of client satisfaction than structural attributes. Long waiting time was association with lower levels of client satisfaction. More client satisfaction was observed in the private sector than in the public sector.
Assaf 2015[49]	Client’s education: no education (AOR= 2.1), primary and post primary (AOR=2.0), provider’s years of education: 6-12 years (AOR=2.9), 13-16years (AOR=3.4) facility region: Dakar (AOR=4.8), Thies (AOR=2.5), central (AOR=11.5), South (AOR=13.9)		Client left with FP methods (AOR=3.7), No counselling on methods side effects (AOR=2.6), counselling on when to return (AOR=2.0), No waiting time (AOR=5.4)	Client age, payment for services, client status, types of contraceptive method used, provider’s job description, provider salary, counselled on how to use the method, health facility type, general structure equipment composite index	The effectiveness of the different forms of counselling was not seen in the outcomes of client overall satisfaction.
Argago 2015[48]	Repeated client (AOR=3.04), history of side effect (AOR=0.121), history of unintended pregnancy (AOR=2.8)	Less than 30min to reach the services (AOR= 5.5), convenient opening hour (AOR=4.73), perceived health facility unclean (AOR= 0.192)	Clients who were advised on how to use the method (AOR=3.43) privacy ensured (AOR= 5.08)	Parity, still birth, number of living children, respect and courtesy, giving written information, told about the methods side effects	The frequency of FP visit, waiting time, cleanness of health facilities, history of side effect, history of unintended pregnancy, and information on how to use methods, privacy during examination and procedure and convenience of opening hour were the predictors of client satisfaction.
Nasr 2016[54][50]		Waiting place^$^, cleanliness of examination room^$^, quality of FP methods^$^, availability of methods^$^ Cost^$^	privacy during examination^$^ waiting time^$^,	Confounders not controlled	The number of received training program affects quality of family planning counselling of nurse’s practice, providers of the services and the provided services affect the client satisfaction.

AOR- Adjusted Odds Ratio β1- Regression coefficient for linear regression analysis β2 = regression coefficient for logistic regression analysis FP-Family Planning H- Hospital (analysis done for hospitals/health centres) C- Clinic/other facilities (analysis done for client/other facilities)

* the factors included only significant factors adjusted for confounders.

** Information given about the method and the time spent for consultation

*** clinic site is easy to get and short waiting time

^$^ p- value for chi-square less than 0.05

The presence of supervision of health facilities in the past six months before the survey from higher officials including from the district federal officials was identified by two studies [20, 38] as a factor determining quality of care in family planning services. However, the two studies presented inconsistent findings with regard to how supervision affected quality of care. Wang et al. [22] showed health facility supervision was positively associated with client satisfaction in two countries, whereas Hutchinson et al. [50] found a negative association between presence of facility’s supervision and client satisfaction. Infrastructure in the form of availability of electricity, water, toilets, and waiting areas were associated with client satisfaction in some levels of health facilities in Kenya and Tanzania [50]. Other structural factors associated with client satisfaction were availability of trained providers, and use of incentives and promotions for health providers [41,50]. While following family planning protocol was positively associated with client’s satisfaction in Tanzania, having client cards/ records was negatively associated with client satisfaction [50]. Costs of family planning services were associated with client satisfaction in two studies [41,54] (Table 2).

#### Process factors

The study conducted in Egypt [47], identified client-provider interaction in the form of positive talk by the provider, as positively associated with client satisfaction. Similarly, client centered family planning services, client’s choosing on the type of contraceptive method to use were more likely to result in client satisfaction [47]. Confidentiality assurance and maintaining privacy were also shown to be positively associated with client’s satisfaction in four studies [41,48,50,54].

Three studies found that information given to the client during the counseling such as information about how to use the method was associated with for client satisfaction [48,50,55]. The studies have presented mixed findings about information provision about side effects as a factor determining quality of care in family planning services [49,50]. The provider’s technical competency was associated with client’s satisfaction in two of the studies [41,50]. In this regard, clients who were asked more reproductive health related questions and underwent physical examination were more likely to be satisfied with the services [41,50]. Hutchnison et al. study [50] measuring the quality of family planning services in three different countries and the study by Wang et al. [22] conducted in for Kenya, showed that injectable method provisions/prescriptions were more likely to bring higher levels of client satisfaction. Counseling on when to return/follow up dates was identified as a factor in two studies [41,49].

Finally, with respect to the results from the quantitative studies, the overwhelming majority of studies (6/8), identified waiting time as a factor determining quality of care in family planning services [22,41,49,50,54,55].

### Synthesized findings on perceptions of factors associated with the quality of care in family planning services from qualitative findings

#### Client perceptions

The limited findings from the two existing qualitative studies [51,52] that have explored how African clients perceive the factors determining quality of care in family planning services, were summarized in three synthesized findings (Table 4).

**Table 4:** Synthesis of qualitative findings on how clients perceive factors determining the quality of care in family planning services.

Findings	Category	Synthesized findings
Participants identified proximity to facility and cost as important considerations for choosing a source, the mode of travel and time to source were never mentioned directly as reasons for choosing a facility.	1. Proximity of services influenced access	***Accessibility of services was important to clients*.** *Proximity to clients’ residing areas*, *costs incurred to get the services*, *convenience of opening hours*, *availability of clients’ preferred contraceptive methods*, *pre-requisites for getting contraceptives were identified by clients as factors that influence service accessibility*.
Proximity was stated as a reason for choice of service delivery points in two ways. Sometimes the respondents gave it as the sole reason for choice or in a combination with other reasons.		
From the combination of reasons for which choice is made, it is clear that proximity is a facilitating factor but not sufficient to sustain use at a health facility.		
Participants identified proximity to facility and cost as important considerations for choosing a source, the mode of travel and time to source were never mentioned directly as reasons for choosing a facility.	2. Cost of services influenced clients choice of facilities/ access	
Among the private clinics, the clients were also able to rank facilities according to the cost of services.		
Though clients complain about cost, they recognize the higher quality of services at Non-Governmental health facilities.		
Women reported that private facilities offered long and convenient service hours that accommodated women’s busy schedules. One woman explained that public facilities often closed before they attended to everyone.	3. Clients tend to prefer facilities having convenient opening hours	
Some women said that they had wasted time waiting at the public facilities for free services, only to find that their preferred method was not available. One woman began to obtain her contraception at a private facility when she found that public facilities did not stock all methods consistently	4. Availability of preferred method (method mix)	
*…*discontinued from Makuyu Health Centre when she found she was not given the injectable, the method that she had wanted. The secondary reason is that the providers asked her to return to the clinic when she was on menses - to make sure that she was not pregnant.	5. Administrative issues in terms of putting pre-requisites for taking contraceptive influences access for family planning services	
Another woman explained that she chose a private facility because she wanted to bypass obstructive processes that she foresaw at the public facility. She had planned to obtain the contraceptive implant at a public facility during her six-week postpartum visit. However, when she received her period four weeks after delivery, she opted for a private facility.		
Women explained that workers at private facilities always provided whichever method was requested. One woman complained that nurses at the public facility prevented her from switching to the injectable contraceptive, so she went to a private facility where they administered her desired method.	1. Responsiveness: Respect for client’s needs and freedom to choose was identified as a factor client’s access to family planning services	***The way care was provided in family planning services was central to clients’ notions of quality care***. Clients identified six qualities of the way care was provided as influencing quality: 1) responsiveness of providers to clients’ self-perceived needs and freedom to choose the contraceptive method; 2) length of waiting time; 3) behavior of providers towards clients; 4) provision of information and support during consultation; 5) privacy and confidentiality; and 6) range of services.
When you walk to a private clinic, you will tell them that you need an injection and when you walk there asking for an injection that is what you will be given.		
… the high degree of dissatisfaction with methods and lack of provider responsiveness to the clients' problems and needs.		
While public facilities were able to provide a broad overview of side effects, they were not able to provide individualized attention. Due to crowded facilities in public healthcare settings, some women were not given the opportunity to address problems with their current method. One woman described her disappointment about not receiving adequate counseling from a public facility when she returned with irregular vaginal bleeding		
Woman in the individual interviews said they preferred public facilities when they needed more decision-making support or guidance for initial selection of a contraceptive method.		
….what irritates clients is when they think the providers are idling while they wait….	2. Waiting time to receive family planning services related with quality of care	
The private sector clinics have a better image with respect to waiting time.		
….they would like family planning services to be provided within an hour of their arrival so that they could get back to their homes quickly before their absence is noticed.		
Even though family planning services were free at the public hospitals, one woman explained that she was willing to pay for contraception at private facilities to avoid waiting in long lines:		
In the public institutions complaints are mostly related to provider behavior while those from the private clinics tend to be related to structural constraints of facilities.	3. Provider behaviour while talking to the client identified as a barrier for quality of care	
Respectful treatment was an added benefit of private facilities. Women believed that private facilities treated their customers with care and attention compared to public facilities where participants experienced verbal harassment, inattention, and rudeness. Respectful behaviour included answering questions kindly and allowing sufficient time for each client. One woman described how rude behaviour at public facilities drove clients to private clinics		
…Though the providers in public institutions are talked of negatively, it should also be pointed out that there are some of them well commended by clients.		
….Sometimes the client is just told to use a certain method and she accepts.	4. Information provision and support in reaching a decision was identified as an important aspect in the delivery of family planning services	
Because of concern for side effects, almost every woman described an ideal family planning visit as one with ample counseling about side effects and support from the provider to choose a method that minimized side effects		
Focus groups participants noted that the private facilities prioritized profit over providing safe medical treatment. While some women mentioned that private providers at non-governmental organization (NGOs) answered questions fully, most women said that most private facilities did not provide counseling or decision support when administering a method.		
While public facilities were able to provide a broad overview of side effects, they were not able to provide individualized attention. Due to crowded facilities in public healthcare settings, some women were not given the opportunity to address problems with their current method. One woman described her disappointment about not receiving adequate counseling from a public facility when she returned with irregular vaginal bleeding		
Woman in the individual interviews said they preferred public facilities when they needed more decision-making support or guidance for initial selection of a contraceptive method.		
Privacy and confidentiality also came up when the topic of client home visits was raised.	5. Maintaining the privacy and confidentiality of clients during family planning provision was valued by clients.	
Women said they used private facilities when they required more confidentiality. One woman related a story of a friend who chose to receive family planning at a private facility to hide her use from her husband.		
Medical examinations were identified by both clients and providers as an important component of family planning service provision which affects choice, continuation and satisfaction with services.	6. Range of services including eligibility screening, blood tests, and physical assessment was valued by clients	
While public facilities were able to provide a broad overview of side effects, they were not able to provide individualized attention. Due to crowded facilities in public healthcare settings, some women were not given the opportunity to address problems with their current method. One woman described her disappointment about not receiving adequate counseling from a public facility when she returned with irregular vaginal bleeding.		
An important factor for the recipient of services was the age and maturity of the providers.	1. Clients perceived to receive family planning services from older and matured provider	***Provider characteristics were identified by clients as factors influencing quality of care*.** These were provider competency and age.
Those who did not mind about the sex of the provider were more concerned about the knowledge and skill of the provider	2. Provider competency in terms of adequate knowledge and skill was valued by clients.	
In individual interviews, women elaborated on their perceptions of the deficiencies in private facilities, which included questionable medications, poor eligibility screening, poorly qualified staff, and poor quality counseling.		
Other women were concerned about the competency of private facility providers. This woman explained her concerns about private providers and her preference for well-qualified public providers		
While it was expected that private facilities would provide a consistent stock of contraceptive supplies, women worried that these facilities administered fraudulent and expired medications to unaware clients. A few women stated that private facilities were more likely to stock expired contraceptives because their inventory exceeded their client flow. This woman attributed two incidences of failed contraception to fraudulent medication provided at private facilities		

**Synthesized finding one: *Accessibility of services was important to clients*. *Proximity to clients’ residing areas*, *costs incurred to get to services*, *convenience of facility’s opening hours*, *availability of clients preferred contraceptive methods*, *and pre-requisites for getting contraceptive methods were identified by clients as factors that influence service accessibility and in turn quality of care*.** Five categories of findings informed this synthesized finding (see Table 4). Category one included findings that spoke about physical proximity of the facilities [52]; category two included findings that were about costs incurred to receive the services [52]. The third category related that convenience, and more specifically opening hours, made services easily accessible [51]. For example, one client said: “I can go [to the private hospital] at any time” [51]p3. The fourth category spoke about clients’ preference for facilities possessing their preferred contraceptive methods [51].

The fifth category included findings which related that pre-requisites expected to be fulfilled by the clients [51,52] affected access. The following client voices illustrate how client’s perceived pre-requisites to be fulfilled such as the requirement of being on menses to take contraceptive methods

When my periods came [at 4 weeks], I felt like it was an emergency, and I didn’t want to waste more time because, like I mentioned, these men are unpredictable and they might demand for it [sex] at any time. I had planned on going for the [public] clinic, but when my menses came I asked a friend if they will allow me to take up family planning at the clinic [early] and she told me that they cannot accept. That is why I went for the method at a private health facility. (Age 27, 3 children, page 4)[51]p4.

**Synthesized finding two: *The way care was provided in family planning services was central to clients’ notions of quality care in family planning services*. *Clients identified six characteristics of the way care was provided as central to their notions of quality of care in family planning services*. *Positive service delivery qualities*, *that clients related they valued*, *and as influencing quality of care included*: *1) responsiveness of providers to clients’ self-perceived needs and freedom to choose the contraceptive method; 2) length of waiting time; 3) behavior of providers towards clients; 4) provision of information and support in making decision during consultation; 5) privacy and confidentiality; and 6) range of services*.** Six categories of findings informed this synthesized finding. Each identified a different attribute of the way care was provided from clients’ perspectives influenced the quality of care. Category one related that clients valued facilities in which providers listened to clients’ needs and allowed them to choose their preferred method [51,52]. Category two included findings related to the time spent before receiving family planning services. Clients indicated that they valued facilities with short waiting time [51,52]. The third category included findings in which clients spoke about provider behavior. Clients said that respectful behaviour of providers was important to them [51,52]. In this regard, a client said that “"…the last time I found a Kisii lady. her advice was good, she was polite like a fellow woman. She showed some signs of respect to me…” [52]p42. The fourth category included findings [51,52] that were about information provision as a factor determining quality of care. Clients said that they valued being provided with information about the side effects of contraception and having their questions adequately responded [51]. The importance of privacy and confidentiality was identified by the findings in category five. One client said that she perceived confidentiality to be facilitated by one to one counseling [51,52]. In the sixth category informing this synthesized finding, clients indicated that a wide range of services, including eligibility screening, blood tests and physical examinations were factors influencing quality of care in family planning services [51,52].

**Synthesized finding three: *Clients perceive two characteristics of health care providers*, *provider competency and provider age as influencing the quality of care in family planning services*.** Two categories informed this synthesized finding. The first was comprised of one finding [52] in which clients related that they preferred older providers because they felt self-conscious about showing their naked body to a young provider. The second category included findings in which clients related they perceived the knowledge and competency of the provider to be a factor determining the quality of care. In this regard, one client said “…the doctor might be qualified, but he could be using his wife to assist him, but the wife is not qualified” [51]p4.

#### Provider perceptions

The very limited qualitative evidence identified, on how providers perceive the factors determining quality of care in family planning services, also generated by two studies [52,53] was summarized in two synthesized findings (Table 5).

**Table 5:** Synthesis of qualitative findings on how providers perceive factors determining the quality of care in family planning services.

Findings	Categories	Synthesized findings
Like the clients, the providers believed that their clinics were chosen partly because of their competitive fees.	1. Cost incurred for family planning services was identified as barriers for family planning services	***Providers identified a range of factors related to the availability of resources*** *including clients’*, low income levels, and inability to pay high fees; inadequate supplies; and high staff workload.
Perceptions of clients’ ability to pay for services influenced the type of care providers offered. Sometimes providers would not bother to make referrals for contraceptive methods or medical treatment if they believed that financial support was lacking.		
Lack of supplies was the most commonly cited barrier to quality family planning services. The few providers who reported that they had enough contraceptive supplies still said they lacked disinfectant, gloves, family planning cards and educational materials. Some stock-outs of contraceptives and other supplies were reported to last 3–6 months and led to discontinuation.	2. Lack of family planning supplies (equipment’s, contraceptive methods, and other materials) were perceived as barriers for family planning services provision	
Providers and managers agreed that many family planning clinics did not stock implants and intrauterine devices because they lacked trained providers who could insert them. Furthermore, lack of training resulted in some providers imposing menstruation barriers–meaning a client must be menstruating before starting a contraceptive method–because they were concerned about inadvertently giving a method to a pregnant woman. Managers agreed this practice occurred and admitted this could result in unintended pregnancies.		
Almost all providers felt that the quality of care they could offer was compromised because they were overloaded with work, and managers confirmed some clinics were understaffed.	3. Workload of providers was identified as factor quality of care in family planning services	
…..the providers were critical of some of their inconsiderate actions at the clinics….	1. Provider behaviour while interacting with clients was identified as a factor for quality of care	***Behavior of providers towards clients***, *for example being disrespectful of client’s time*, *and privacy and confidentiality of clients for example the need to hide from husbands and parents were identified as factors affecting the quality of care*. *The range of services offered by providers to clients was also identified as factor influencing the quality of care*.
Providers reported that many women secretly used contraceptive methods. A woman who hides use and experiences a side effect is at risk of stopping the method rather than switching to a method that might be detected by her husband, they said. Informed choice loses much of its meaning when the primary use criterion is a method that cannot be detected.	2. Privacy and confidentiality for client from other clients and parents were factor for quality of care	
Medical examination were identified by both clients and providers as an important component of family planning service provision which affects choice, continuation and satisfaction with services.	3. Providers perceived range of services such as conducting medical examination as an important element in the provision of family planning services	

**Synthesized finding four: *Providers identified factors related to the availability of resources as influencing the quality of care in family planning services*. *These included*: *clients’ low income levels*, *and inability to pay high fees; inadequate supplies in facilities; and high staff workload*.** Three categories informed this synthesized findings. The first category related that the high costs of family planning services relative to clients’ income undermined clients’ ability to choose facilities and methods, and affected quality of care [52,53]. Category two informed that lack of family planning supplies often prevented providers from delivering the type of services clients intended to receive [53]. The high workload of providers in the facility influenced the delivery of quality of care for their family planning clients [53] was the third category. The following two illustrations were client’s voices supporting the second and third categories. In the words of one provider: “*We had no Depo-Provera, for a long time … over 6 months actually. [Question: So what were you doing by that time?] Those who wanted Depo were not being served because most people here do not like oral contraceptives.” (Manager)[53]p38*. The third category included only one finding which identified too few staff relative to clients as a factor affecting quality of care [53]. In the words of a provider: “*We are overloaded. We are small doctors [laughter]. There are clients for Voluntary Counselling and Testing (VCT) there … in the labour ward; there are two mothers waiting and then there are clients in antenatal clinic … If these mothers wait for so long, they have to go elsewhere and if they do not get their method they will never come back.” (Bushenyi, FGD) [53]p39.*

**Synthesized finding five: *Behavior of providers towards clients*, *for example being disrespectful of client’s time*, *regard for privacy and confidentiality for family planning clients*, *were perceived by providers as factors affecting the quality of care*. *Providers also identified the range of services offered by providers to clients as a factor influencing the quality of care*.** This synthesized finding was informed by three categories of findings. The first described provider behavior as something that affected quality of care [52,53]. The second spoke the importance of privacy and confidentiality of clients affecting quality of care [53]. The third identified the range of services as a factor affecting quality [52]. The following provider voice illustrates that the providers perceptions about clients need about range of services.

"I have seen a client coming from town [to our clinic] and she already was provided with pills but she said she was not examined. She told her friend who told her that one normally is supposed to be examined…So I think they value examinations—general examinations and pelvic" (INDEPTH, Provider 11, Location 2) [52]p44.

## Discussion

This systematic review aimed to develop an evidenced based understanding of factors that determine the quality of care in family planning services in Africa, using a systematic review of mixed evidence. A total of 11 moderate to high quality studies, undertaken in seven of the fifty five African countries, were identified and included to inform understanding of the factors determining quality of care in family planning services in Africa.

The quantitative component of the review pointed to a wide range of factors determining the quality of care in family planning services in Africa. These included client, provider, facility, structural and process factors. Client’s waiting time before receiving the services, provider competency, provision of injectable methods, maintaining privacy and confidentiality were the most commonly identified process factors found in the quantitative studies. Quality of stock inventory was the most commonly identified structural factor identified by the quantitative evidence. In addition, the quantitative studies pointed to the type of ownership of facilities as an important factor influencing quality of care in family planning services. More specifically, privately owned facilities were associated with higher levels of client satisfaction than publicly owned facilities. We also found from the quantitative studies, that certain client characteristics, namely age, educational status, and types of clients in terms of being whether new or repeat users were associated with satisfaction with family planning services.

A number of factors that were identified by the quantitative studies as important factors determining the quality of care in family planning services were mirrored in the synthesized findings from the included qualitative studies. These were waiting time, information provided to clients, maintaining client’s privacy and assurance of confidentiality, provider competency, convenient operating hours and cost of family planning services. The qualitative findings pointed towards three additional factors not identified by the quantitative studies. These were pre-requisites to be fulfilled by clients before taking family planning services, workload of providers, and provider behaviour.

The findings from the systematic review corroborate the aspects of quality of care of family planning identified in the Bruce and Jain framework [28,30]. The aspects that the Bruce and Jain Framework suggested, were mostly related to the process of family planning services provision included the contraceptive method mix, the information provided to the clients, provider’s technical competency, the interpersonal relationship between provider and clients, continuity and follow-up, and constellation of services [28,30]. However, the current review highlighted additional factors that were associated with quality of care. Firstly, service accessibility issues including cost of services and the presence of certain pre-requisites such as clients were required to confirm they were on their menses at the time of contraceptive initiation. Secondly, waiting time and client’s preference for older and mature providers were identified as additional factors determining quality of care in family planning services from the qualitative studies. The range of factors identified by this review as influencing the quality of care in family planning services in Africa points to the limitations for the Donabedian model in the sense that it excludes the patient’s own characteristics as important factors determining quality of care [29]. In this regard, the included studies in the current review showed age and educational status of clients as factors associated with quality of care.

Similar to this review, a review of studies conducted in the United States, showed quality of family planning services as depending on a range of factors including the characteristics of the facility, provider, and client [56]. Three studies, one in United States [57] and two [58,59] conducted in Iran, also highlighted access to services as an important factor influencing quality of care in family planning services.

This systematic review has several strengths and shortcomings. The first strength is its inclusion of qualitative and quantitative studies. The inclusion of mixed evidence in systematic review is widely regarded as best practice [60,61] to provide decision makers with information to inform policy making as qualitative evidence can aid understanding, by offering insights into factors that cannot be measured. A second strength of this reviews is the consideration and inclusion of academic (peer reviewed) and grey literature. Inclusion of grey literature addresses the issue such as publication bias [62]. The use of best practice method for systematic review, and in particular the conduct of critical appraisal and formal synthesis of qualitative findings using meta-aggregation is a third strength.

This systematic review also has a number of limitations. Firstly, the included quantitative studies employed different definitions classification and measurement approaches of client satisfaction and hence meta-analysis was not possible. Secondly, the studies were conducted in a small number of Africa’s diverse countries and regions. The findings may reflect health service structures and client/provider perceptions that are specific to these areas and may not be applicable to other African countries and regions. Thirdly, there were relatively few quantitative studies and very few qualitative studies included in our review. These last two limitations may limit the generalizability of the results. However, some factors were found to be significant in all or the majority of the studies, which suggests that these may be applicable to a number of settings. Finally, although client’s perceptions are often mediated through the social and cultural environment [63], the included studies did not consider the cultural aspects of family planning clients.

### Implications of the policy and planning of family planning services in Africa

The limited size and heterogeneous nature of the evidence base identified by this review precluded identifying the factors that are most important, in a wide range of African settings, for the provision of high quality of care in family planning services in Africa. This prevents the drawing of firm evidence based recommendations for health planners wanting to implement measures to improve quality of care in family planning services in all health settings in Africa. However, our findings about the factors influencing quality of care in family planning services do offer some guidance for health planners about strategies that should be priorities. First, in this regard, the positive association found between quality of care and structural factors related to the facility, including proximity to clients’ place of residence, costs of services, and the number of days in a week that the service is open, points towards the need for planners to implement strategies that reduce these access barriers. Towards this end, subsidized or free services, outreach services, flexible opening hours of clinics/hospitals, and arrangements of transportation are options that could be explored. Second, the finding that provider competency is an important factor determining quality of care in family planning services suggests that investing in provider skills, and supporting providers to deliver care in a way that is congruent with best practice, are important. Third, the finding that the provision of information about planning methods is an important factor determining quality of care, suggests that strategies to ensure that clients are supplied with necessary information about the different methods, and their potential side effects are important to support high quality of care in family planning services. Fourth, the findings from our review point to the need for planners to implement strategies to shorten clients’ waiting time and ensure client’s privacy and confidentiality in family planning services.

## Conclusion

Overall, the limited, moderate to high quality, quantitative and qualitative studies on factors determining quality of care in family planning services in Africa, pointed to multiple factors, related to client, provider, facility characteristics, as well as structural and process. Hence, improving quality of care in family planning services in Africa requires multiple actions that target these different factors. Further research is required to understand the key factors associated with quality of care in family planning services in African countries.

## Supporting Information

S1 Fig PRISMA Flow Diagram. (TIF)

S1 Table PRISMA 2009 Checklist. (DOCX)

S2 Table Search strategy. (RTF)

S3 Table Findings and illustrations of the included qualitative studies. (RTF)

S4 Table Methodological quality assessment. (RTF)

S1 Text Records excluded at full text examination with reasons. (RTF)
