# Supplementary figures and images for: Factors Determining Quality of Care in Family Planning Services in Africa: A Systematic Review of Mixed Evidence
Source: PLoS One. 2016 Nov 3;11(11):e0165627. doi: 10.1371/journal.pone.0165627 (PMC5094662; doi:10.1371/journal.pone.0165627)

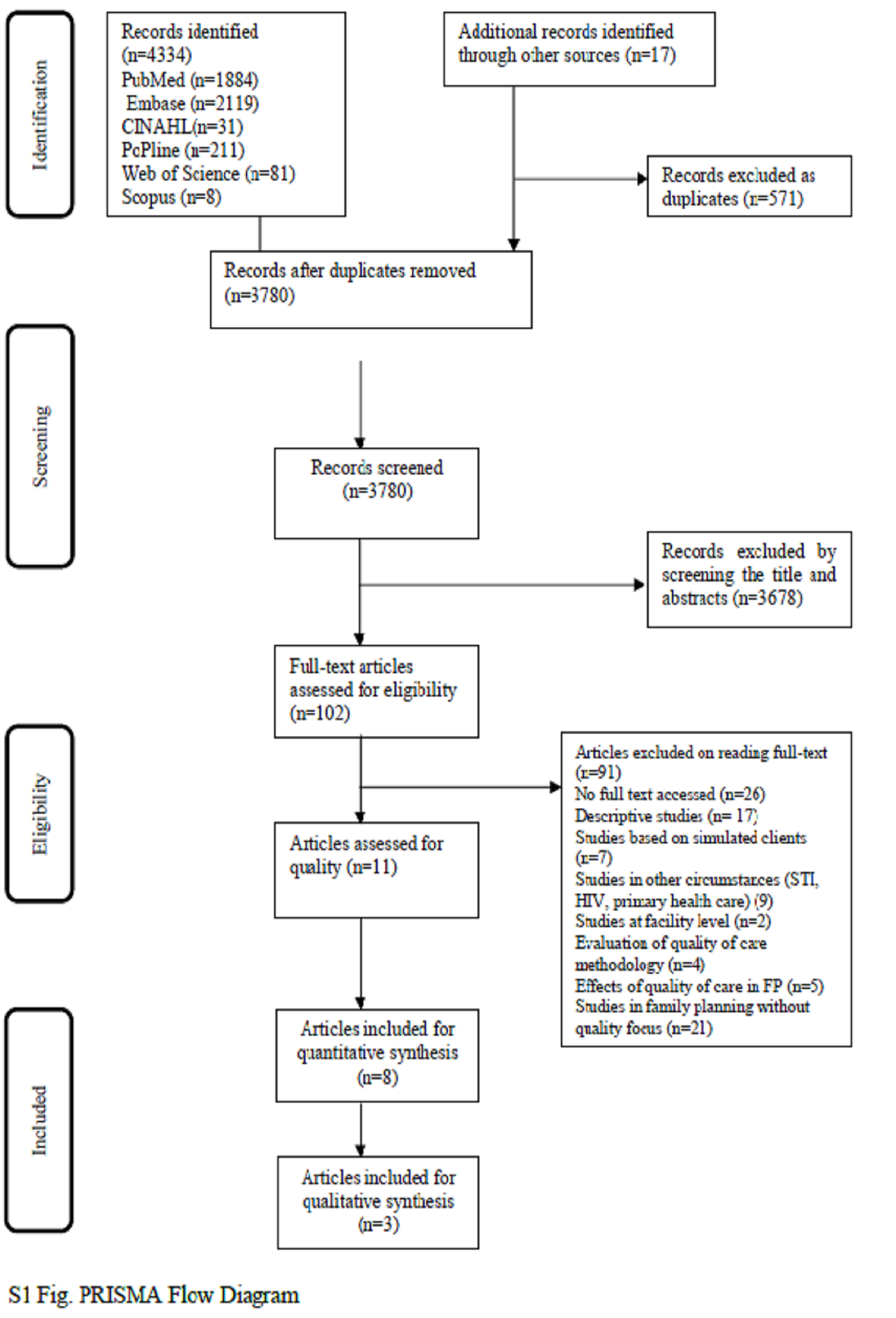

Supplement: S1 Fig — (TIF) [file pone.0165627.s001.tif]
