# Supplementary material for: Factors Determining Quality of Care in Family Planning Services in Africa: A Systematic Review of Mixed Evidence
Source: PLoS One. 2016 Nov 3;11(11):e0165627. doi: 10.1371/journal.pone.0165627 (PMC5094662; doi:10.1371/journal.pone.0165627)
Supplement: S2 Table — (RTF) [file pone.0165627.s003.rtf]

S2 Table. Search strategy 
Quality 	Family planning	Africa 	
PubMed	
Quality of health care[mh] OR Quality of health care[tiab] OR quality of care[tiab] OR quality of healthcare[tiab] OR health care quality[tiab] OR  healthcare quality[tiab] OR quality[tiab] OR Patient satisfaction[mh] OR 
Satisfaction[tiab] OR
Preference*[tiab]	Family planning services[mh]
OR Family planning service*[tiab] OR family planning[tiab] OR contraceptive service*[tiab] OR contraception service*[tiab] OR birth control service*[tiab] OR fertility control service*[tiab] OR contraception[mh] OR 
Pregnancy control*[tiab] 


	Africa[mh] OR Africa*[tiab] OR Algeria*[tiab] OR Angola*[tiab] OR Benin*[tiab] OR Botswana*[tiab] OR Burkina Faso [tiab] OR Burundi*[tiab] OR Cape Verde*[tiab] OR  Cabo Verde [tiab] OR Cameron*[tiab] OR Cameroon*[tiab] OR Chad*[tiab] OR Comoros*[tiab] OR Congo*[tiab] OR Cote d'Ivoire[tiab] OR Ivory coast [tiab] OR Djibouti*[tiab] OR Egypt*[tiab] OR Eritrea*[tiab] OR Ethiopia*[tiab] OR Gabon*[tiab] OR Gambia*[tiab] OR Ghana*[tiab] OR Guinea*[tiab] OR Kenya*[tiab] OR Lesotho*[tiab] OR Liberia*[tiab] OR Libya*[tiab] OR Madagascar*[tiab] OR Malawi*[tiab] OR Mali*[tiab] OR Maurit*[tiab] OR Morocc*[tiab] OR Mozambiqu*[tiab] OR Namibia*[tiab] OR Niger*[tiab] OR Rwanda*[tiab] OR Senegal*[tiab] OR Seychelles[tiab] OR Sierra Leone*[tiab] OR Somalia*[tiab] OR Sudan*[tiab] OR Swaziland*[tiab] OR Tanzania*[tiab] OR Togo*[tiab] OR Tunisia*[tiab] OR Uganda*[tiab] OR Zambia*[tiab] OR Zimbabwe*[tiab]	
Embase 	
'health care quality'/exp OR 'health care quality':ti,ab OR 'healthcare quality':ab,ti OR 'quality of health care':ab,ti OR 'quality of healthcare':ab,ti OR 'patient satisfaction'/de OR 'satisfaction'/exp OR 'satisfaction':ab,ti OR 'preference':ab,ti	'family planning'/exp OR 'family planning':ab,ti OR 'contraceptive service':ab,ti OR 'contraceptive services':ab,ti OR 'birth control service':ab,ti OR 'birth control services':ab,ti OR 'contraception service':ab,ti OR 'contraception services':ab,ti OR 'fertility control services':ab,ti OR 'contraception'/exp	Africa/exp OR Africa*:ti,ab OR Algeria*:ti,ab OR Angola*:ti,ab OR Benin*:ti,ab OR Botswana*:ti,ab OR 'Burkina Faso':ti,ab OR 'Burkina Fasso':ti,ab OR Burundi*:ti,ab OR 'Cape Verde':ti,ab OR 'Cape Verdean':ti,ab OR 'Cabo verde':ti,ab OR Cameron*:ti,ab OR Cameroon*:ti,ab OR Chad*:ti,ab OR Comoros*:ti,ab OR Congo*:ti,ab OR 'Cote dIvoire':ti,ab OR  'Ivory coast':ti,ab OR Djibouti*:ti,ab OR Egypt*:ti,ab OR Eritrea*:ti,ab OR Ethiopia*:ti,ab OR Gabon*:ti,ab OR Gambia*:ti,ab OR Ghana*:ti,ab OR Guinea*:ti,ab OR Kenya*:ti,ab OR Lesotho*:ti,ab OR Liberia*:ti,ab OR Libya*:ti,ab OR Madagascar*:ti,ab OR Malawi*:ti,ab OR Mali*:ti,ab OR Maurit*:ti,ab OR Morocc*:ti,ab OR Mozambiqu*:ti,ab OR Namibia*:ti,ab OR Niger*:ti,ab OR Rwanda*:ti,ab OR Senegal*:ti,ab OR Seychelle*:ti,ab OR 'Sierra Leone':ti,ab OR 'Sierra Leonean':ti,ab OR Somalia*:ti,ab OR Sudan*:ti,ab OR Swaziland*:ti,ab OR Tanzania*:ti,ab OR Togo*:ti,ab OR Tunisia*:ti,ab OR Uganda*:ti,ab OR Zambia*:ti,ab OR Zimbabwe*:ti,ab	
CINAHL	
MH “quality of health care” OR  TI "quality of health care" OR AB “quality of health care” OR  TI "quality of healthcare" OR AB “quality of healthcare” OR TI "healthcare quality" OR AB "healthcare quality" OR TI "health care quality" OR AB "health care quality" OR MH “patient satisfaction” OR TI “satisfaction” OR AB “satisfaction”	MH “family planning” OR 
TI “family planning” OR AB “family planning” OR TI  “contraceptive service*” OR AB  “contraceptive service*” OR 
TI “birth control service*” OR AB “birth control service*” OR AB  “fertility control service*” OR TI “contraception service*” OR AB  “contraception service*” OR MH 'contraception'	MH Africa OR TI Africa* OR AB Africa* OR TI Algeria* OR AB Algeria* OR TI Angola*OR AB Angola* OR TI Benin*OR AB Benin* OR TI Botswana* OR AB Botswana OR TI “Burkina Faso*” OR AB “Burkina Faso*” OR TI Burundi* OR AB Burundi* OR TI “Cape Verde*” OR AB “Cape Verde*” OR TI Cameron* OR AB Cameron*OR TI Cameroon*OR AB Cameroon* OR TI Chad* OR AB chad* OR TI Comoros* OR AB Comoros* OR TI Congo* OR AB Congo* OR TI “Cote d'Ivoire” OR AB  “Cote d'Ivoire” OR TI “Ivory coast” OR AB “Ivory coast” OR TI Djibouti* OR AB Djibouti* OR TI Egypt* OR AB Egypt* OR TI Eritrea* OR AB Eritrea* OR TI Ethiopia* OR AB Ethiopia* OR TI Gabon* OR AB Gabon* OR TI Gambia*OR AB Gambia* OR TI Ghana* OR AB Ghana OR TI Guinea* OR AB Guinea* OR TI Kenya* OR AB Kenya* OR TI Lesotho* OR Lesotho* OR TI Liberia* OR AB Liberia OR TI Libya* AB Libya* OR TI Madagascar* OR AB Madagascar* OR TI Malawi* OR AB Malawi* OR TI Mali* OR AB  Mali* OR TI Maurit* OR AB Maurit* OR TI Morocc* OR AB Morocc* OR TI Mozambiqu* OR AB Mozambiqu* OR TI Namibia* OR AB Namibia* OR TI Niger* OR AB Niger* OR TI Rwanda* OR AB Rwanda* OR TI Senegal* OR AB Senegal* OR TI Seychelles* OR TI Seychelles* OR TI “Sierra Leone*” OR AB “Sierra Leone*” OR TI Somalia* OR AB Somalia” OR TI Sudan* OR AB Sudan* OR TI Swaziland* OR AB Swaziland* OR TI Tanzania* OR AB Tanzania* OR TI Togo* OR AB Togo* OR TI Tunisia* OR AB Tunisia OR TI Uganda* OR AB Uganda* OR TI Zambia* OR TI Zambia* OR TI Zimbabwe* OR AB Zimbabwe*	
Scopus 			
"Quality of health care" OR "quality of healthcare" OR "health care quality" OR "healthcare quality" OR "quality of care" Or "quality" OR "Satisfaction" OR Preference	“family planning” OR “family planning servce*” OR“contraceptive service*” “birth control service*” OR “fertility control service*” OR “contraception service*” OR 'contraception'	Africa OR Algeria* OR Angola* OR Benin* OR Botswana*OR “Burkina Faso” OR Burundi* OR “Cape Verde*” OR  “Cabo Verde” OR Cameron* OR Cameroon*  OR Chad* OR Comoros* OR Congo* OR “Cote d'Ivoire” OR “Ivory coast” OR Djibouti* OR Egypt* OR Eritrea* OR Ethiopia* OR Gabon* OR Gambia* OR Ghana* OR Guinea* OR Kenya* OR Lesotho* OR Liberia* OR Libya* OR Madagascar* OR Malawi* OR Mali* OR Maurit* OR Morocc* OR Mozambiqu* OR Namibia*OR Niger*OR Rwanda* OR Senegal* OR Seychelles OR Sierra Leone* OR Somalia* OR Sudan* OR Swaziland* OR Tanzania* OR Togo* OR Tunisia* OR Uganda* OR Zambia* OR Zimbabwe*	
POPLINE  	
 Quality OR (“Quality of care”) OR (“quality of health care”) OR (“healthcare quality”) OR (“health care quality”) 	 (“family planning”) OR (“Family planning services”) OR (“contraceptive services”) OR contraception OR  (“fertility control”) OR (“contraception services”) OR 'contraception'	Africa	


 Date limiter:  Date:  01/01/1990; language: English 
Date of search: April 30, 2016
