# Supplementary material for: Factors Determining Quality of Care in Family Planning Services in Africa: A Systematic Review of Mixed Evidence
Source: PLoS One. 2016 Nov 3;11(11):e0165627. doi: 10.1371/journal.pone.0165627 (PMC5094662; doi:10.1371/journal.pone.0165627)
Supplement: S3 Table — (RTF) [file pone.0165627.s004.rtf]

S3 Table. Findings and illustrations of the included qualittative studies 
Lewis N. Quality of care in family planning service delivery in Kenya: Clients' and providers' Perspective 1995.
Finding: ….. Participants identified proximity to facility and cost as important considerations for choosing a source, the mode of travel and time to source were never mentioned directly as reasons for choosing a facility.
Illustration: "Actually, the reason I had gone to Murang'a hospital is because it was near and free" (FGD 1, Switcher, Location 1) page 33
Illustration:" I chose Maragua Rural Health Center because it was the nearest service delivery point. You see, I come from Kiriani where the providers used to bring a mobile family planning clinic from Muririnjas [they stopped] so Maragua is the nearest service delivery point." (FGD 6, Current, Location 2) page 33	
Finding: Proximity was stated as a reason for choice of service delivery points in two ways. Sometimes the respondents gave it as the sole reason for choice or in a combination with other reasons.
Illustration: "You should start with the location, it should be well located.. It was not easily accessible and the number of clients who used to come there used to be very few. When it rained, the paths were not accessible.. The location should be number one" (Indepth, Provider 8, Location 2). Page 33
Illustration: "I think one thing it has to be near and the transport has to be accessible, it has to be clean and then the relationship between the staff and the client in general" (Indepth, Provider 5, Location 1). Page 33.	
Finding 3: From the combination of reasons for which choice is made, it is clear that proximity is a facilitating factor but not sufficient to sustain use at a health facility.
Illustration: "There is a clinic very near to where I live but I used to hear bad stories about it." (FGD 2, Current, Location 1). Page 33	
Finding: Among the private clinics, the clients were also able to rank facilities according to the cost of services.
Illustration: "Yes, the thing I can say is that their charges are very low and because that clinic is only for family planning, they are very good at family planning"(FGD 5, Switcher, Location 1) page 34
Illustration: "When we take our children to the City Council, we do not pay for the services, but if you go to a private clinic, you must pay" (FGD 1, Current, Location 1). Page 34	
Finding: Though clients complain about cost, they recognise the higher quality of services at Non-Governmental health facilities. Serah represented this point of view in her wish as….
Illustration: "I would like to change this clinic because of the 20 shillings that is required per visit. Actually if I find another clinic with as good services as this one and it is not charging I would change to that "(FGD 2, Current, Location 1). Page 34	
Finding:  Like the clients, the providers believed that their clinics were chosen partly because of their competitive fees. 
Illustration: " ...like we used to have so many clients but when we increased the charges slightly from 10 shillings to thirty some of them stopped coming here. They were saying that the charges were so high... They also don't mind paying because the charges are very minimal compared to the services we offer" (Indepth, Provider 8, Location 2). Page 34	
Finding: …..the providers were critical of some of their inconsiderate actions at the clinics….
Illustration:"The long waiting time. We come and start chatting - giving stories and the clients are waiting and nobody is attending to them" (Indepth, Provider 2, Location 1).page 36	
Finding: ….what irritates clients is when they think the providers are idling while they wait….
Illustration:" I would like to spend just about half an hour, that would be my wish. But the providers have other things to prepare before they call clients.. so in consideration for this the providers should be given time to prepare" (FGD 6, Current, Location 2). page 37
Illustration: "I have not experienced that but what I don't like is the slow attendance. They make us wait for too long in the queue and that time we are so many. You see the doctors are concentrating on their talks and others are sleeping and when you go in .. they send you out" (FGD 5, Switcher, Location 1). Page 37	
Finding: The private sector clinics have a better image with respect to waiting time. 
Illustration: " There are some people who might be discouraged because of the congestion here.. they prefer to go to private clinics where they will not be kept waiting"(INDEPTH, Switcher 1, Location 1) page 38	
Finding: ….no woman sought family planning services before the birth of a child. 
Illustration: "I started thinking about family planning in 1979 when I had my second child. Actually, they were too close together as I got pregnant when my first child was only six months.. so I made a decision to space my children" (FGD 6,Current, Location 2). Page 38	
Fining: ….Sometimes the client is just told to use a certain method and she accepts.
Illustration: ".. I did no go back to the clinic in July, 1989 but September 1989. The provider told me this was the best method and I decided to use it [the coil]".(current user) page 39
Illustration: "A friend of mine told me that the injection was the best and I should use it "(FGD 4, Discontinuer, Location 1). Page 39	
Finding: Privacy and confidentiality also came up when the topic of client home visits was raised.
Illustration: The clients are shy and need privacy.. I would like to emphasize that one to one counselling is important ". (family planning user) page 40	
Finding: Both clients and providers dwelt extensively on the importance of friendly and pleasant relations at the clinics.
Illustration: "I want to be properly welcome at the clinic, I would like to feel wanted and look like a bother" (FGD, Current, Location 1) page 41	
Finding: …Though the providers in public institutions are talked of negatively, it should also be pointed out that there are some of them well commended by clients.
Illustration: "..The last time I found a Kisii lady.. her advice was good, she was polite like a fellow woman.. she showed some signs of respect to me. That is what I like in that clinic (INDEPTH, Discontinuer 1, Location 1) page 42	
Finding: This group of women argued that as long as the provider had mastered family planning knowledge and skills, the basic qualification such as nursing/midwifery and medicine did not matter.
Illustrations:  "…doctors are too busy and therefore in too much of a hurry to finish each case and go the next".	
Finding:  An important factor for the recipient of services was the age and maturity of the providers. 
Illustration: ……" I have heard women saying that they will not go to seek family planning services because the providers are younger than themselves and they have no wish to show their nakedness to young providers" (FGD 6, Current, Location 2). Page 43	
Finding: …both clients and providers as an important component of family planning service provision which affects choice, continuation and satisfaction with services.
Illustration: "The provider checks whether the coil is in situation. Then urine is sent to the laboratory to exclude infections. I appreciate this service" (FGD 10, Current, Location 3) page 44   
Illustration: "I have seen a client coming from town [to our clinic] and she already was provided with pills but she said she was not examined. She told her friend who told her that one normally is supposed to be examined...So I think they value examinations - general examinations and pelvic" (INDEPTH, Provider 11, Location 2).	
Finding: … the high degree of dissatisfaction with methods and lack of provider responsiveness to the clients' problems and needs.
Illustration: "I went and found that the doctor was there but people were not being attended to, there was no injection but those for pills were being given. Then I went to look for a place to buy the injection and I couldn't get it. .. I went home but the next day I went looking for the injection but I stayed for about one week because I was very annoyed of what they had done to me. It was then that I went and bought the injection but on going back to the clinic, I was told to wait, they do a check-up whether I am pregnant - things like that. So I felt bad and from that time I decided that I will not go back so I switched." (Indepth, Switcher 9 , Location 4) page 46	
Finding: …discontinued from Makuyu Health Centre when she found she was not given the injectable, the method that she had wanted. The secondary reason is that the providers asked her to return to the clinic when she was on menses - to make sure that she was not pregnant.
Illustration: " I was not satisfied with the pill. I took it for about 9 months. I used to feel weak, nauseous. I could not work and so I felt that I couldn't continue using something which could not help me and so I decided to stop." (Indepth, Discontinuer 6, Location 3). Page 48	
Finding: ….While depo Provera received positive comments, the use of IUCD seemed to be fraught with unpleasant experiences…
Illustration: "I used to bleed a lot and having lower abdominal pains. I also used to have watery discharge and a lot of itching...This itching also gave me sores. I thought that it was the IUCD which was giving me all these problems. I went to the general hospital to have it removed but anytime I went there, they used to have so many problems and telling me that it couldn't be removed. I saw a lady friend of mine who told me that she would bring me here to Marie Stopes. She brought me and on that first day it was removed. After explaining to them my problems, they promised that they would remove it. I couldn't walk straight because of the pain I had. I have not had any problem with the pill." (Indepth, Switcher 5, Location 2) page 49.	


Keesara SR, Juma PA, Harper CC. Why do women choose private over public facilities for family planning services? A qualitative study of post-partum women in an informal urban settlement in Kenya. BMC Health Serv Res. 2015;15:335.
Finding: Even though family planning services were free at the public hospitals, one woman explained that she was willing to pay for contraception at private facilities to avoid waiting in long lines:
Illustrations: There are many private clinics around here that some women prefer to go to because if you go to Mathare North dispensary [public facility] you will have to queue for long. Some women prefer to pay 100 KES at the private clinic and be over with it (Focus Group Participant). Page 3	
Finding: Women reported that private facilities offered long and convenient service hours that accommodated women's busy schedules. One woman explained that public facilities often closed before they attended to everyone.
Illustration: I can go [to the private hospital] at any time. At public health facilities they take long to give service. Some people would wait and even give up and go back home, or some don't even feel like going there because you would go in the morning and leave at 1:00… So some opted to go to private health facilities because if you wanted an injection you would just walk there and pay then receive the injection, and walk out, and they don't take a lot of your time and you can go and do other things. (Age 32, 3 children). Page 3	
Finding: Some women said that they had wasted time waiting at the public facilities for free services, only to find that their preferred method was not available. One woman began to obtain her contraception at a private facility when she found that public facilities did not stock all methods consistently
Illustration: I have only used the injection and pills. I used to buy the pills at the chemist [pharmacist] so I didn't have to come to the hospital. The first time I started using pills, when I went to the hospital, they didn't have pills and I had to buy from the chemist so I continued buying from the chemist. (Age 38, 4 children)	
Finding: Women explained that workers at private facilities always provided whichever method was requested. One woman complained that nurses at the public facility prevented her from switching to the injectable contraceptive, so she went to a private facility where they administered her desired method
Illustration: I used pills after my first delivery but within the first two days I really got sickly and I stopped using them. I threw them away because I had headaches, I didn't feel like doing anything. I went to the [public] hospital they told me that I should persevere and finish the dose. I got upset and I threw them away and went to the private clinic and went for the injection. (Age 34, 2 children). Page 3	
Finding:. Another woman explained that she chose a private facility because she wanted to bypass obstructive processes that she foresaw at the public facility. She had planned to obtain the contraceptive implant at a public facility during her six-week postpartum visit. However, when she received her period four weeks after delivery, she opted for a private facility
Illustration: …When my periods came [at 4 weeks], I felt like it was an emergency, and I didn't want to waste more time because, like I mentioned, these men are unpredictable and they might demand for it [sex] at any time. I had planned on going for the [public] clinic, but when my menses came I asked a friend if they will allow me to take up family planning at the clinic [early] and she told me that they cannot accept. That is why I went for the method at a private health facility. (Age 27, 3 children) page 4	
Finding: Respectful treatment was an added benefit of private facilities. Women believed that private facilities treated their customers with care and attention compared to public facilities where participants experienced verbal harassment, inattention, and rudeness. Respectful behaviour included answering questions kindly and allowing sufficient time for each client. One woman described how rude behaviour at public facilities drove clients to private clinics:
Illustration: For instance if I was using a method and it was not working for me, then I come back to the hospital and the nurse starts yelling at me like, “You woman, don't be foolish.” You know such things are making many women go to the private facilities because when you go there people respect you. They should respect us and address us like adults and not insult us. (Focus group participant) page 4	
Finding: Women said they used private facilities when they required more confidentiality. One woman related a story of a friend who chose to receive family planning at a private facility to hide her use from her husband
Illustration: Her husband didn't want her to take family planning but he was not giving her any valid reasons why she shouldn't take that up, so she just went privately. It was difficult, but she went to a chemist so that she can be able to go when the husband is not around. (Age 24, 2 children) page 4.	
Finding: Focus groups participants noted that the private facilities prioritized profit over providing safe medical treatment. While some women mentioned that private providers at non-governmental organization (NGOs) answered questions fully, most women said that private most facilities did not provide counseling or decision support when administering a method.
Illustration: In private clinics it's about money. They don't have time to counsel you. They don't offer good services. By the time you go to the clinic, you have a method in mind, so they just administer. (Focus Group Participant).	
Finding: In individual interviews, women elaborated on their perceptions of the deficiencies in private facilities, which included questionable medications, poor eligibility screening, poorly qualified staff, and poor quality counseling.
Illustration: When you visit the private health facilities, they will just ask you what method you want and they won't offer counseling services. They will not even ask how old the baby is, they will give you what you went for. They will just ask you when you had your last menses and go ahead and maybe you are pregnant they don't run tests, they will just inject you. (Age 23, 2 children) page 4	
Finding: Other women were concerned about the competency of private facility providers. This woman explained her concerns about private providers and her preference for well-qualified public providers
Illustration: In public facilities the doctors are qualified but in private facilities it could be a quack, or the doctor might be qualified, but he could be using his wife to assist him, but the wife is not qualified. But in public facilities you always find qualified staff from the doctor to all the other employees. So their services are genuine and you don't get scared when they are attending to you. (Age 34, 4 children) page 4	
Finding: While it was expected that private facilities would provide a consistent stock of contraceptive supplies, women worried that these facilities administered fraudulent and expired medications to unaware clients. A few women stated that private facilities were more likely to stock expired contraceptives because their inventory exceeded their client flow. This woman attributed two incidences of failed contraception to fraudulent medication provided at private facilities
Illustration: Some women are injected with water and you keep feeling safe that you have used family planning while you are unsafe and you eventually you end up pregnant. That has happened to two women. One had gone to the chemist and another one at a private facility. They ask you for money but they don't render the services. (Age 23, 1 child) page 4	
Finding: Because of the concern for poor quality medical treatment at private facilities, some women said that they preferred to endure long waits at public facilities: 
Illustration: After that story I had heard from those women about private hospitals, I was reluctant to do it [go to private providers] because maybe their medication for family planning is also expired, I was comparing them to the chemists because they are all private businesses. And so I decided to be patient and go to the Government clinics (Age 25, 3 children) page 5	
Finding: woman in the individual interviews said they preferred public facilities when they needed more decision-making support or guidance for initial selection of a contraceptive method. This woman explained her decision to seek services at public facilities to decide about her method of contraception after delivery:
Illustration: I got more information from the [Nairobi] City Council hospitals. Private [hospitals] don't have time for such talk [counseling about family planning]. They are more concerned about their time. So after delivery, regardless of where I have delivered I visit [Nairobi] City Council clinics and they provide training on that. (Age 34, 3 children) page 5	
Finding: When you walk to a private clinic, you will tell them that you need an injection and when you walk there asking for an injection that is what you will be given.
Illustration…They don't do any tests to establish whether you should have used the pills or coil or Norplant and then you end up developing side effects. (Focus Group Participant)	
Finding: Because of concern for side effects, almost every woman described an ideal family planning visit as one with ample counseling about side effects and support from the provider to choose a method that minimized side effects
Illustration: A good family planning visit is whereby when you enter the room you are counseled first, then you get to choose one and she tells you the side effects and she recommends what you should use suiting your body and not just allowing you to go for a method you had already decided to use even if it's not good. (Age 21, 1 child) page 5	
Finding: Minimization of side effects was one of the highest priorities when choosing a contraceptive method. While public facilities were able to provide a broad overview of side effects, they were not able to provide individualized attention. Due to crowded facilities in public healthcare settings, some women were not given the opportunity to address problems with their current method. One woman described her disappointment about not receiving adequate counseling from a public facility when she returned with irregular vaginal bleeding
Illustration: I expected her to counsel me more about family planning, but the first thing she did when she met us was whine that we were late. She only asked me what method I wanted and I told her the injection and that was it. (Age 23, 1 child) page 5	

Mugisha JF, Reynolds H. Provider perspectives on barriers to family planning quality in Uganda: a qualitative study. Journal of Family Planning & Reproductive Health Care. 2008;34(1):37-41 5p.
Findings:  Lack of supplies was the most commonly cited barrier to quality family planning services. The few providers who reported that they had enough contraceptive supplies still said they lacked disinfectant, gloves, family planning cards and educational materials. Some stock-outs of contraceptives and other supplies were reported to last 3–6 months and led to discontinuation.
Illustrations: “We had no Depo-Provera, for a long time … over 6 months actually. [Question: So what were you doing by that time?] Those who wanted Depo were not being served because most people here do not like oral contraceptives.” (Manager) (page 38)	
Finding:   Almost all providers felt that the quality of care they could offer was compromised because they were overloaded with work, and managers confirmed some clinics were understaffed. Individual providers take on multiple responsibilities such as antenatal care, labour and delivery, voluntary HIV counselling and testing (VCT) and childhood vaccinations, in addition to family planning services. The work overload was complicated by the tendency of large numbers of clients to visit clinics on open market days and immunisation days.
Illustration: “We are overloaded. We are small doctors [laughter]. There are clients for VCT there … in the labour ward; there are two mothers waiting and then there are clients in antenatal clinic … If these mothers wait for so long, they have to go elsewhere and if they do not get their method they will never come back.” (Bushenyi, FGD)	
Finding: Providers and managers agreed that many family planning clinics did not stock implants and intrauterine devices because they lacked trained providers who could insert them. Furthermore, lack of training resulted in some providers imposing menstruation barriers–meaning a client must be menstruating before starting a contraceptive method–because they were concerned about inadvertently giving a method to a pregnant woman. Managers agreed this practice occurred and admitted this could result in unintended pregnancies.
Illustration: “To me, some providers believe that once a client is not in her menses they should go back [home without contraceptives].” (Manager) page 39
Illustration:  “[The client] said that 'last time I went to the health facility for family planning and they told me to come back when I am in my menses, by the time I went back I was already pregnant!'” (Manager) page 39	
Finding: Providers reported that many women secretly used contraceptive methods. A woman who hides use and experiences a side effect is at risk of stopping the method rather than switching to a method that might be detected by her husband, they said. Informed choice loses much of its meaning when the primary use criterion is a method that cannot be detected. 
Illustration:  “They come and get services except they may want a method which we don't have. Now this lady is escaping from the husband. Then you tell her go to Jinja and does not get counselled, she will not come back.” (Iganga, FGD page 39)	
Finding:  Perceptions of clients' ability to pay for services influenced the type of care providers offered Sometimes providers would not bother to make referrals for contraceptive methods or medical treatment if they believed that financial support was lacking. 
Illustration: “... goes back home and she conceives because she cannot afford the method she wanted”. (Mpigi, FGD) page 39 	
Finding: Providers were frustrated with continually being faced with clients' misconceptions, which included associating contraceptives with infertility, loss of manhood (in the case of vasectomy), loss of libido, disability, menstrual blood accumulating in the body, and fibroids, among others. Providers reported that these misconceptions were, in part, propagated by politicians and the media, especially radio campaigns that promulgated negative attitudes about family planning, and were thought by providers to cause many clients to discontinue family planning methods.
Illustration: “[I] do not think that there is political will. The top leadership of Uganda has never talked about family planning and its benefits.” (Manager) page 39	
