# Supplementary material for: Factors Determining Quality of Care in Family Planning Services in Africa: A Systematic Review of Mixed Evidence
Source: PLoS One. 2016 Nov 3;11(11):e0165627. doi: 10.1371/journal.pone.0165627 (PMC5094662; doi:10.1371/journal.pone.0165627)
Supplement: S4 Table — (RTF) [file pone.0165627.s005.rtf]

S4 Table.  Methodological quality assessment 
First author, year of publication and reference number  	Q1	Q2	Q3	Q4	Q5	Q6	Q7	Q8	Q9	Q10	Overall quality of the study 	
Critical appraisal for included quantitative studies 	
Tewab et al. 2002[36]	N	Y	Y	Y	Y	N	Y	Y			6/8 (moderate)	
Agha et al. 2009[37]	N	Y	Y	Y	U	N	Y	Y			5/8(moderate)	
Hutchnison et al. 2011[40]	N	Y	Y	Y	Y	N	Y	Y			6/8 (moderate)	
Tafese et al. 2013[45]	Y	Y	Y	Y	Y	N	Y	Y			7/8 (high) 	
Wang et al. 2014[22]	N	Y	Y	Y	Y	N	Y	Y			6/8(moderate)	
Argago et al. 2015[38]	Y	Y	Y	Y	U	N	Y	Y			6/8(moderate)	
Assaf et al. 2015[39]	N	Y	Y	N	Y	N	Y	Y			5/8(moderate)	
Nasr et al. 2016[44]	N	Y	N	Y	Y	Y	Y	Y			6/8(moderate)	
Number of studies that achieved compliance	2	8	7	7	6	1	8	8				
Critical appraisal for included qualitative studies	
Ndhlovu et al. 1995[42]	N	Y	Y	Y	Y	N	N	Y	N	Y	6/10(moderate)	
Mugisha et  al. 2008 [43]	N	Y	Y	Y	Y	N	N	Y	Y	Y	7/10 (high) 	
Keesara et al. 2015[41]	N	Y	Y	Y	Y	N	N	Y	Y	Y	7/10 (high) 	
Number of studies that achieved compliance	0	3	3	3	3	0	0	1	1	1		
Criteria was adapted from the JBI Critical Appraisal Checklist for descriptive/case series research[31]. For quantitative studies: (1) Was the study based on a random or pseudo-random sample? (2) Were the criteria for inclusion in the sample clearly defined? (3) Were confounding factors identified and strategies to deal with them stated? (4) Were outcomes assessed using objective criteria? (5) If comparisons were being made, was there sufficient description of the groups? (6) Were the outcomes of people who withdrew described and included in the analysis? (7) Were outcomes measured in a reliable way? (8) Was appropriate statistical analysis used? For qualitative studies: (1) Was there congruency between the stated philosophical perspective between the research and the methodology? (2) Was there congruity between the research methodology and the research question or objectives? (3) Was there congruity between research methodology and data collection methods (4) Were there congruity between research methodology and representation and analysis of data (5) Were there congruity between research methodology and interpretation of results? (6) Was there a statement locating the researcher culturally or theoretically? (7) Was the influence of the research on the research and vice versa addressed? (8) Were participants and their voices adequately represented? (9)Was the research ethical according to current criteria or, for recent evidence of ethical approval by an appropriate body? (10) Did the conclusions drawn in the research report flow from the analysis or interpretation of the data? Each item was rated Y = Yes, N = No or U = Unclear. Unclear was awarded where not enough information was provided. High quality: meets ≥ 7 criteria, Moderate quality: meets ≥ 4 criteria, Low quality: < 4 criteria.  
