# Supplementary material for: Factors Determining Quality of Care in Family Planning Services in Africa: A Systematic Review of Mixed Evidence
Source: PLoS One. 2016 Nov 3;11(11):e0165627. doi: 10.1371/journal.pone.0165627 (PMC5094662; doi:10.1371/journal.pone.0165627)
Supplement: S1 Text — (RTF) [file pone.0165627.s006.rtf]

S1 Text: Records excluded at full text examinations with reasons
1.	Agha, S. et al: The impact of a quality-improvement package on reproductive health services delivered by private providers in Uganda. Studies in Family Planning 41(3):205-215
Reason for exclusion: it assessed the effectiveness of quality improvement packages. It did not identify the potential factors affecting the quality of care in FP 
2.	Ali MM: Quality of care and contraceptive pill discontinuation in rural Egypt. J Biosoc Sci. 2001;33(2):161-72
Reason for exclusion: the study aimed to see the effects of quality of care [quality of care was deemed as exposure of interest] 
3.	Askew et al: Quality of care in family planning programmes: A rapid assessment in Burkina Faso.  Health Policy and Planning1993:8(1): 19-32
Reason for exclusion: methodological assessment 
4.	Baumgartner, J. N. et al: Service delivery characteristics associated with contraceptive use among youth clients in integrated voluntary counseling and HIV testing clinics in Kenya. AIDS Care - Psychological and Socio-Medical Aspects of AIDS/HIV 24(10): 1290-1301
Reason for exclusion: it looked the FP integration with the HIV program. The outcome of interest was not client satisfaction. 
5.	Brown, L: Quality of care in family planning services in Morocco. Studies in family planning 1995, 26(3)154-168
Reason for exclusion: descriptive study without identifying the factors 
6.	Daff, B et al: Informed push distribution of contraceptives in Senegal reduces stockouts and improves quality of family planning services. Global health sciences and Practice 2014, 2(2):245-252
Reason for exclusion: it assessed the contribution of early distribution of family planning commodities but did not assess the quality of care wither from users or providers perspective. 
7.	Dusabe, J. et al:  "There are bugs in condoms": Tanzanian close-to-community providers' ability to offer effective adolescent reproductive health services. J Fam Plann Reprod Health Care 2015, 41(1)e2
Reason for exclusion: TBAs were not our population of interest
8.	Fantahun M: Quality of family planning services in Northwest Ethiopia 2005, 19(3): 195-200
Reason for exclusion: descriptive study focusing on what exists in terms of quality. It did not identify what affects quality of care in family planning services. 
9.	Finocchairo-kessker: "I may not say we really have a method, it is gambling work": knowledge and acceptability of safer conception methods among providers and HIV clients in Uganda. Health care for women international2014, 35(7-9): 896-917.
Reason for exclusion: this study focusses on the alternative approaches of contraception in HIV patients. Did not assess quality of care 
10.	Gilson, L: The structural quality of Tanzanian primary health facilities. Bull World health organ 1995: 73(1): 105-14
Reason for exclusion: Assessed quality from facilities point of view. The analysis is not at clients. 
11.	Hailemariam A, Quality of Care in the Delivery of Family Planning Services in Ethiopia: A baseline assessment at selected service delivery facilities of two non-governmental organizations FHI Addis Ababa 1999.
Reason for exclusion: this is a descriptive study that did not examine the potential factors for quality of care.
12.	Hancock, NL Evaluation of service quality in family planning clinics in Lusaka, Zambia. Contraception, 92 (4): 345-9
Reason for exclusion: descriptive study that did not identify the factors.
13.	Hebert, L. E et al Family planning providers' perspectives on family planning service delivery in Ibadan and Kaduna, Nigeria: a qualitative study J Fam Plann Reprod Health Care, 2013: 39 (1), 29-35
Reason for exclusion: it looked into the barriers of family planning use but not on specific notion of quality of care in the services
14.	Global network of people living with HIV: Quality of family planning services and integration in the prevention of vertical transmission context 2014 
Reason for exclusion: it conducted in three counties assessed the integration of family planning in HIV care; not the factors related to quality of care 
15.	Hong R eta al: Family planning services quality as a determinant of use of IUD in Egypt. BMC Health Serv Res 6: 79 
Reason for exclusion: it assessed the effect of quality of care in family planning, not on what affects the quality of care itself.
16.	Huntington, D et al: User's perspective of counselling training in Ghana: the "mystery client" trial Stud fan plan 1990, 21(3): 171-177
Reason for exclusion: This was both a descriptive study and based on mystery clients 
17.	Kakoko, DC et al Provision of family planning services in Tanzania: a comparative analysis of public and private facilities. Afr J Rep Health 2012, 16 (4): 140-148
Reason for exclusion: it compared quality of care at facilities level not at client's level.
18.	Kelner, A: Perceptions of clients regarding family planning service delivery in a clinic of the Greater Johannesburg Metropolitan Council Curtionis 2010 33(2): 13-24
Reason for exclusion: it has just look into whether women's expectations in family planning were met or not. It lacks information on the factors about quality of care.
19.	Khamis, K et al: Patients' level of satisfaction on quality of health care at Mwananyamala hospital in Dar es Salaam, Tanzania. BMC Health serv res 2014, 14:8
Reason for exclusion: it looked into the quality of care delivered at primary care setup. It is general and did not target family planning 
20.	Leon, FR et al Quality of delivery of the Standard Days Method as compared with contraceptive pills in Rwanda. J family planning and rep health care 2006, 32(4): 231-233
Reason for exclusion: simulated clients study 
21.	Letaief, M: Implementing a quality improvement programme in a family planning centre in Monastir, Tunisia Eastern Mediterranean Health Journal 2008 14(3):11
Reason for exclusion: descriptive assessment for some aspects of quality of care
22.	Loha E, Assessment of quality of care in family planning services in Jimma Zone, Southwest Ethiopia. 2003, 18(1): 8-18
Reason for exclusion: descriptive study 
23.	Maharaj, P et al: The quality of integrated reproductive health services: perspectives of clients in KwaZulu-Natal, South Africa. Curationis 2005, 28(1) 52-58
Reason for exclusion: it has focused on the level of integration of family planning with other services.
24.	Mancici DC et al: The effect of structural characteristics on family planning program performance in Cote d'Ivoire and Nigeria
Reason for exclusion: this study examine what factors influence family planning services provision but not a specific component of family planning. 
25.	Marlow, HM et al Postpartum Family Planning Service Provision in Durban, South Africa: Client and Provider Perspectives. Health Care for Women International 2014, 35(2) 175-199
Reason for exclusion: it focused on family planning use, not on its quality component.
26.	McHome, Z. et al: A 'mystery client' evaluation of adolescent sexual and reproductive health services in health facilities from two regions in Tanzania. PloS One, 10(3),e0120822
Reason for exclusion: it was based on Mystery client's that were not real family planning clients in addition to the fact that they envisaged adolescents experiences in reproductive health services not limited to family planning services. 
27.	McKenna, K. et al:  Policy and programmatic considerations for introducing a longer-acting injectable contraceptive: perspectives of stakeholders from Kenya and Rwanda. Glob Health Sci Pract 2014, 2(4), 459-71
Reason for exclusion: it focused on single family planning method; not on the whole family planning services  
28.	Mekonnen G, Prevalence and factors affecting use of long acting and permanent contraceptive methods in Jinka town, Southern Ethiopia: a cross sectional study. Pan afr med J, 18: 98
Reason for exclusion: This study identified factor for family planning rather than quality of services. 
29.	Melaku Y et al: Sexual and reproductive health communication and awareness of contraceptive methods among secondary school female students, northern Ethiopia: a cross-sectional study. BMC Public Health, 2014, 14;252
Reason for exclusion: it was a family planning study but not related with quality of care.
30.	Mensch, B: Using situation analysis data to assess the functioning of family planning clinics in Nigeria, Tanzania, and Zimbabwe. Studies in family planning 1994, 25(1): 18-31
Reason for exclusion: This study is a descriptive study that did not identify the factors affecting quality of care.
31.	Miller RA: The Situation Analysis Study of the family planning program in Kenya. Studies in family planning 1991, 22(1):131-143
Reason for exclusion: descriptive study
32.	MLE project: Trends in family planning service quality in Kisumu, Kenya: A Research Brief. 2013
Reason for exclusion: descriptive study
33.	Mohamed, SS: Involvement of Sudanese community pharmacists in public health activities. International Journal of Clinical Pharmacy, 2013, 35(3):393-400
Reason for exclusion: This studies focusing on describing the roles of community workers
34.	Mohan, Y et al: Providing family planning services to remote communities in areas of high biodiversity through a Population-Health-Environment programme in Madagascar. Reproductive Health Matters, 2014, 22(43): 93-103
Reason for exclusion: This study provided information on the importance of integrating family planning with other program. It did not assess the factors associated with quality of care 
35.	Mroz, TA: Quality, accessibility, and contraceptive use in rural Tanzania. Demography, 1999, 36(1):23-40
Reason for exclusion: This study assessed the effects of quality of care on contraceptive use 
36.	Muhammed, KA: Understanding the barriers to the utilization of primary health care in a low-income setting: Implications for health policy and planning. Journal of Public Health in Africa, 2013, 4(2): 64-67
Reason for exclusion: Focused on primary health care services
37.	Murashami, J: Quality of Family Planning Services in HIV/AIDS Care and Treatment Clinics in Tanzania. University of North Carolina, USA, 2013
Reason for exclusion: Descriptive study
38.	Mwaniki MK: Improving service uptake and quality of care of integrated maternal health services: the Kenya kwale district improvement collaborative. BMC health serv research, 2014, 14:9
Reason for exclusion: It assessed antenatal care services; not family planning services 
39.	Myburgh, NG et al: Patient satisfaction with health care providers in South Africa: the influences of race and socioeconomic status. International Journal for Quality in Health Care, 2005, 17(6), 473-477
Reason for exclusion: Assessed general patient satisfaction in the whole health services 
40.	Nalwadda, G: Quality of care in contraceptive services provided to young people in two Ugandan districts: a simulated client study. PLoS One, 6(11):e27908
Reason for exclusion: used simulated clients
41.	Nguyen, H, Assessing public and private sector contributions in reproductive health financing and utilization for six sub-Saharan African countries. Reproductive Health Matters, 19(37): 62-74
Reason for exclusion: did not assess quality of services 
42.	Obare F, Community-level effect of the reproductive health vouchers program on out-of-pocket spending on family planning and safe motherhood services in Kenya. BMC health serv res 2015, 15:343
Reason for exclusion: did not assess quality of services 
43.	Olowu F: Quality and costs of family planning as elicited by an adolescent mystery client trial in Nigeria. African journal of reproductive health 1998, 2(1): 49-60
Reason for exclusion: used mystery clients besides its descriptive nature 
44.	Pitorak, H: "It Depends on Your Pocket:" Findings From a Qualitative Study in Uganda Exploring Women's and Health Care Providers' Perspectives on Family Planning. Health Care for Women International, 2014, 35(3),234-248
Reason for exclusion: it focused on family planning services not specific to quality of care 
45.	RamaRao, S. The quality of family planning programs: Concepts, measurements, interventions, and effects. Studies in Family Planning, 2003, 34(40): 227-248
Reason for exclusion: descriptive study and did not identify factor 
46.	Sanogo, D. et al: Improving quality of care and use of contraceptives in Senegal. African journal of reproductive health. 7(2): 57-73
Reason for exclusion: descriptive study 
47.	Shabangu, NP: The quality of family-planning services in Swaziland. Africa Journal of Nursing & Midwifery 2005, 7(1): 23-29
Reason for exclusion: descriptive study
48.	Shah, NM: Comparing private sector family planning services to government and NGO services in Ethiopia and Pakistan: how do social franchises compare across quality, equity and cost? Health policy and planning, 2011, 26suppl1: i63-71
Reason: did not identify the factors 
49.	Sieverding, M: User experiences with clinical social franchising: qualitative insights from providers and clients in Ghana and Kenya. BMC Health Serv Res, 2015, 15:49
Reason: Explored social franchising network; the phenomenon of interest did not much 
50.	University of Southampton: Quality of family planning services in Malawi: What can exit interviews tell us. Fact Sheet 16 Reproductive Health Research funded by DFID, 
Reason: descriptive study 
51.	Speizer, IS: Demand generation activities and modern contraceptive use in urban areas of four countries: a longitudinal evaluation. Glob Health Sci Pract, 2014,2(4): 410-26
Reason: the outcome of interest was not quality of care 
52.	Stein, K: Using situation analysis to assess women's perception of quality of maternal-child health and family planning services. Reproductive Health Matters, 1998, 6(11):45-54
Reason: methodological assessment 
53.	Sullivan, TM: Skewed contraceptive method mix: why it happens, why it matters. J Biosoc Sci, 2006, 38(4), 501-21
Reason: it did not assess quality of care 
54.	Tapsoba,P: Quality of care and client willingness to pay for family planning services at Marie Stopes International in Burkina Faso. 2013, Ouagadougou, Burkina Faso
Reason: descriptive study 
55.	Tavrow, P: Measuring the quality of supervisor-provider interactions in health care facilities in Zimbabwe. International Journal for Quality in Health Care. 2002, 14(1): 57-66
Reason: not specific to family planning services 
56.	Thatte, N: Does human resource management improve family planning service quality? Analysis from the Kenya Service Provision Assessment 2010. Heakth policy plan, 2015, 30(3): 356-67
Reason: the outcome variable was not client satisfaction
57.	Thamas, JC: Integration opportunities for HIV and family planning services in Addis Ababa, Ethiopia: an organizational network analysis. BMC Health Serv Res, 2014, 14:22
Reason: did not assess quality of care 
58.	Tumlinson, K: Simulated clients reveal programmatic factors that may influence contraceptive use in Kisumu, Kenya. Glob Health Sci Pract 2013, 1(3): 407-416
Reason: it was based on simulated clients 
59.	Tumlinson K: Quality of Care and Contraceptive Use in Urban Kenya. International Perspectives on Sexual & Reproductive Health, 2015, 41(2), 69-79
Reason: quality of care was taken as independent variable; not as an outcome of interest 
60.	Tumlinson, K: Accuracy of standard measures of family planning service quality: findings from the simulated client method. Stud fam Plann, 2014, 45(4): 443-70
Reason: methodological assessment 
61.	Tuoane M, Use of Family Planning in Lesotho: The Importance of Quality of Care and Access. African Population Studies. Year## 18:2 
Reason: client satisfaction was not the outcome for assessing quality of care
62.	Ujuju C: An assessment of the quality of advice provided by patent medicine vendors to users of oral contraceptive pills in urban Nigeria. J Multidiscip Healthc, 2014, 7: 163-71
Reason: the study was conducted using simulated clients
63.	Vahdat, HL: There are some questions you may not ask in a clinic: providing contraception information to young people in Kenya using SMS. Int J Gynaecol Obstet 2013, 123(1): e2-6
Reason: did not assess quality of care
64.	Van EL: The challenge of measuring quality of care at health centre level in Africa: The example of Tsholotsho health district in Matabeleland North, Zimbabwe. International Journal of Health Planning and Management 2007, 22(1): 63-89
Reason: client satisfaction was not the outcome of interest  
65.	Westaway, MS: Satisfaction with family planning services: interpersonal and organisational dimensions. Curationis 1998, 21(4): 3-7: 
Reason: methodology assessment study
